# Transcriptomic, metabonomic and proteomic analyses reveal that terpenoids and flavonoids are required for *Pinus koraiensis* early defence against *Bursaphelenchus xylophilus* infection

**DOI:** 10.1186/s12870-025-06192-8

**Published:** 2025-02-12

**Authors:** Lu Yu, Yanna Wang, Xiang Wang, Shan Han, Laifa Wang, Xizhuo Wang

**Affiliations:** 1 Key Laboratory of Forest Protection of National Forestry and Grassland Administration, Ecology and Nature Conservation Institute, Chinese Academy of Forestry, Beijing, 100091 China; 2 Chinese Society of Forestry, Beijing, 100091 China; 3 College of Forestry, Sichuan Agricultural University, Sichuan, 611130 China

**Keywords:** Pine wilt disease, Pine wood nematode, Plant resistance, Responses to biotic stresses

## Abstract

Pine wilt disease (PWD), caused by the pine wood nematode (PWN) *Bursaphelenchus xylophilus*, threatens *Pinus* seriously. *Pinus koraiensis* is one of the most important pine species in China and is the host for PWN. However, our understanding of the defence-regulating process following infection by *B. xylophilus* at the molecular level remains limited. To understand the mechanisms that *P. koraiensis* responds to *B. xylophilus* invasion, *P. koraiensis* was inoculated with *B. xylophilus* solutions and observed no obvious symptoms during the early stage; symptoms began to appear at 5 dpi. Therefore, we conducted comparative transcriptomic, metabonomic and proteomic analyses between *P. koraiensis* 5dpi and 0 dpi. In infected plants, 1574 genes were significantly up-regulated, including 17 terpenoid-, 41 phenylpropanoid- and 22 flavonoid-related genes. According to GO and KEGG enrichment analyses of significantly up-regulated genes, 86 GO terms and 16 KEGG pathways were significantly enriched. Most terms and pathways were associated with terpenoid-, phenylpropanoid-, flavonoid- and carbohydrate-related events. Similarly, the abundance of 36 and 30 metabolites, significantly increased in positive and negative polarity modes, respectively. Among them, naringenin and 3-methyl-2-oxovaleric acid exhibited significant toxic effects on *B. xylophilus*. According to functional analysis of significantly up-regulated metabolites, most terms were enriched in above pathways, in addition to alkaloid biosynthesis. Although the abundance of few proteins changed, response to stress term was significantly enriched in significant up-regulated proteins. Furthermore, plant receptor-like serine/threonine kinases, pectin methylation modulators, pinosylvin O-methyltransferase and arabinogalactan/proline-rich proteins were significantly up-regulated in the infected *P. koraiensis* compared to healthy plants. These proteins were not abundant in the healthy plant. Overall, these results indicate that *P. koraiensis* can actively response to PWN via various defense strategies, including events related to terpenoids, flavonoids, phenylpropanoids, lipids and alkaloids. Particularly, terpenoids and flavonoids are required for the early defence of *P. koraiensis* against *B. xylophilus* infection.

**Supplementary Information:**

The online version contains supplementary material available at 10.1186/s12870-025-06192-8.

## Introduction

Pine wilt disease (PWD) is one of the greatest threats to pine trees and is spreading worldwide [1, 2]. Substantial economic losses from PWD have been reported, and it also threatens the ecosystems of pine forests in countries such as Japan and China [3, 4]. The disease largely comes from the pine wood nematode (PWN) (*Bursaphelenchus xylophilus*), specifically from its vector, the cerambycid beetles (*Monochamus* spp.). *Bursaphelenchus xylophilus* enters pine trees, such as, *Pinus thunbergii*, *Pinus massoniana*, *Pinus pinaster* and *Pinus tabuliformis* [5–9], as the beetles feed on the twigs of these trees. When entering a tree, *B. xylophilus* moves through the resin canals and lives off tissues of plant stem, triggering a progressive blockage of water flow and even the death of the tree [2, 10]. Visible symptoms of PWD include needle chlorosis and wilting [11].

The susceptibility and resistance of trees varies in light of *Pinus* species (interspecific variability) and trees in the same species (intraspecific variability) [12]. Numerous physiological, histological, structural, and biochemical studies have been conducted to explore the defense responses of *Pinus*. A series of chemical changes may occur in infected trees, and various metabolites, such as terpenoids [2] and phytoalexins [13], stilbenoids [14] and flavonoids [14], which are related to plant resistance mechanisms, may also accumulate in the trees [15, 16]. Some of these compounds exhibit nematocidal activity [17].

Recently, plant responses to PWN infection have been investigated at the molecular defense level. Comparative transcriptomic analyses of *Pinus* species with different susceptibilities to *B. xylophilus* infection have revealed different strategies for handling nematode infection [8, 18–20]. For instance, *Pinus pinea*, exhibited a relatively high abundance of genes related to oxidative stress and elevated expression levels of stress-responsive genes [21]. In addition, many recent studies have focused on gene expression changes in *Pinus* species infected by *B. xylophilus* at various time points [6, 22, 23]. Notably, the transcription of chalcone synthase genes in *Pinus thunbergii* and *Pinus massoniana* differed significantly at different time points post-infection, indicating that pine trees might inhibit *B. xylophilus* invasion by activating chalcone synthase expression, leading to the accumulation of phytoalexins [22].

To fully understand resistance mechanisms, it is crucial to integrate transcriptomics with proteomics and metabolomics. This is because differences in mRNA levels may not always correlate with protein abundance or activity due to factors such as post transcriptional regulation, mRNA secondary structures or the availability of cellular resources, and post translational modifications, which influence protein structure or function [24]. Proteomic differences between the two *P. massoniana* of different provenances inoculated with *B. xylophilus* highlight the presence of proteins involved in hydrogen peroxide scavenging, which help maintain redox homeostasis system and are associated with resistance [25]. Another proteomic study on resistant clones of *P. massoniana* inoculated with *B. xylophilus* and revealed highly expressed aspartic proteases, suggesting their role in degrading nematode-related proteins [26]. However, few studies have focused on metabolomic comparisons of host trees with different susceptibilities to *B. xylophilus*. Infection by PWN induces localized changes in the primary metabolite levels of *P. pinaster* [27]. Resistant *P. pinaster* plants collect osmolytes, such as fucose, GABA, trehalose, after inoculation, which play a key role in protecting cells from oxidative damage [27].

After *B. xylophilus* infection, pine trees embrace a significant transcriptional change following with changes in the synthesis of proteins and metabolites. Studies on more resistant pine varieties and families have identified several resistance mechanisms, including reactive oxygen species (ROS) detoxification, cell wall reinforcement, and the production of secondary metabolites, which likely work in concert to confer resistance to *B. xylophilus* [14, 27, 28]. Among these, the synthesis of terpene compounds has been linked to resistance against several pests in pine species [29]. Increased expression of terpene synthase genes was observed in resistant *P. massoniana* in response to *B. xylophilus* infection [19]. The enzymatic products, α-pinene and longifolene were shown to directly reduce the survival rate of *B. xylophilus* in vitro [28]. In addition, flavonoids, which are widely distributed in plants, also play a vital role in plant resistance to infection. Chalcone synthase, a key enzyme in the phenylpropanoid and polyketide pathways, is essential for flavonoid biosynthesis [30, 31]. Pine trees might inhibit PWN invasion by activating chalcone synthase expression, leading to the accumulation of phytoalexins [22].

Since 2016, PWN has rapidly spread into the colder northern regions of China, with confirmed cases of PWD reported in Liaoning Province and Jilin Province, which border Russia and North Korea [7, 32]. The northwards expansion has resulted in distinctive epidemiological characteristics, including latent infections and delayed tree mortality [7, 33, 34]. In some cases, infections remain latent for over a year, during which infected pine trees show no symptoms, making detection challenging and perpetuating the spread of nematodes via insect vectors [7, 35]. Additionally, new host plants and insect vectors, previously absent in areas of China affected by PWD, have been discovered in this region [36]. *Pinus koraiensis*, one of the most important pine species in the region, is widely distributed across southern parts of the Russian Far East, the Korean Peninsula, and Japan [34]. The invasion of PWD into northeastern China poses a serious threat not only to *P. koraiensis* forests in China but also to those in neighbouring regions of Russia and North Korea.

In our previous study, the pathogenicity of *B. xylophilus* in *P. koraiensis* was explored via inoculation of plants with three PWN isolates [7]. However, the molecular response of *P. koraiensis* to *B. xylophilus* infection remains insufficiently understood. Further research is needed to understand the defense mechanisms of *P. koraiensis* against nematode infection. These studies provide a theoretical foundation for developing new control strategies for pine wilt disease in this region. Therefore, in this study, we adopted artificial inoculation to analyze the defense response of *P. koraiensis* at different time points following inoculation with *B. xylophilus*. Moreover, we conducted comparative transcriptomic, metabonomic and proteomic analyses between early infected plants and healthy plants. These findings will benefit the early diagnosis of *B. xylophilus* infection and provide crucial date for further research on the pathogenesis of *B. xylophilus*.

## Materials and methods

### Nematodes and plants

*Bursaphelenchus xylophilus* QH-1, which was extracted from infected *P. koraiensis* in Liaoning, China, was used as a wild-type nematode in this study. Nematodes-cultivation relied on *Botrytis cinerea* on barley grains for two weeks at 25 °C. The nematodes were extracted at room temperature with the help of Baermann funnel technique. The concentration of nematode fluid was 10,000 nematodes per milliliter of water.

A nursery was used to grow *P. koraiensis* seedlings (four-year-old) in the Ecology and Nature Conservation Institute in Beijing, China. The seedlings were transplanted into plastic pots and moved to the nursery in the spring of 2019. The experimental seedlings were domesticated for a few months in order to eliminate the influence from abiotic stress factors.

### Inoculation process

In a greenhouse, holes were drilled into four-year-old potted *P. koraiensis* seedlings at an angle of 45° below the main stem at a location 10–15 cm above the ground, 40 μL of pinewood nematode mixture (250/μL) was infused into the hole with a pipette, which was sealed as soon as possible to avert evaporation. After 1, 5, 10 or 20 days post inoculation with the pine wood nematode mixture, pine needles (approximately 1 g) were taken as experimental sample and were transferred to a 2.0 mL tube for storage. Twenty pine trees were used as replicates for each treatment (infected and healthy *P. koraiensis*).

### Transcriptomic sequencing and analysis

Three trees representing infected and healthy *P. koraiensis* were selected as biological replicates for transcriptomic sequencing. Under the instruction from manufacturer, total RNA was obtained from samples through an RNAprep Pure Plant Kit (Tiangen). Then RNA quality was determined by 5300 Bioanalyser (Agilent) and quantified using the ND-2000 (NanoDrop Technologies). Only high-quality RNA sample (OD260/280 = 1.8 ~ 2.2, OD260/230 ≥ 2.0, RQN ≥ 6.5) was used to construct sequencing library. Then, transcriptome samples were prepared via Illumina kits. All cDNA libraries were sequenced on an Illumina HiSeq platform with 150 bp paired-end reads obtained. In-house Perl scripts were used to deal with those raw reads. The sequencing library was performed on NovaSeq X Plus platform(PE150) using NovaSeq Reagent Kit. Then clean reads were generated through removing reads containing adapter, poly-N and low-quality reads via fastp software (fastp -g -q 5 -u 50 -n 15 -l 150) [37].

Then clean data from the samples were used to do de-novo assembly with Trinity [38]. To increase the assembly quality, all the assembled sequences were filtered by CD-HIT [39] and TransRate [40] and assessed with BUSCO (Benchmarking Universal Single-Copy Orthologs) [41]. The assembled transcripts were searched against the NCBI protein nonredundant (NR), Clusters of Orthologous Groups of proteins (COG), and Kyoto Encyclopedia of Genes and Genomes (KEGG) databases using Diamond to identify the proteins that had the highest sequence similarity with the given transcripts to retrieve their function annotations and a typical cut-off E-values less than 1.0 × 10^−5^ was set. BLAST2GO [42] program was used to get GO annotations of unique assembled transcripts for describing biological processes, molecular functions and cellular components.

The number of reads (counts) on each gene was counted by featureCounts v1.5.0-p3, and quantification of gene expression was on account of FPKM (transcript kilobase fragments per million fragments mapped) values. Genes that were distinctively expressed between the compared groups were determined via DESeq2. R package with the following criteria: |log2 (fold change)|≥ 1; *p* < 0.05. The transcriptome sequence of the wild-type strain was used as the control. The filtered differentially expressed genes (DEGs) were rated by GO and KEGG enrichment analyses via the clusterProfiler R package. GO and KEGG terms with adjusted *p* values < 0.05 were considered significantly enriched with DEGs.

### Metabolomic sequencing and analysis

Three trees representing infected and healthy *P. koraiensis* were selected as biological replicates for metabolomic sequencing.

EP tubes were used to contain samples, then resuspended in prechilled 80% methanol by vortexing. Being whirled (melted on ice for 30 s), sonicated (6 min), and centrifuged (5,000 rpm, 4 °C for 1 min), the supernatants of all the samples were freeze-dried and dissolved in 10% methanol. Finally, the solution was added into an LC–MS/MS system for further study.

UHPLC-MS/MS was for the composition of the solutions analysis in a Vanquish UHPLC system (Thermo Fisher, Germany), combined with an Orbitrap Q ExactiveTM HF mass spectrometer (Thermo Fisher, Germany). The samples were injected onto a Hypesil Gold column (100 × 2.1 mm, 1.9 mm) with a 12-min linear gradient at a flow rate of 0.2 mL/min. The Q ExactiveTM HF mass spectrometer worked in positive/negative polarity mode with a spray voltage of 3.5 kV, capillary temperature of 320 °C, sheath gas flow rate of 35 psi, auxiliary gas flow rate of 10 L/min, S-lens RF level of 60, and auxiliary gas heater temperature of 350 °C.

The original data generated by UHPLC-MS/MS were treated via Compound Discoverer 3.1 (CD3.1, Thermo Fisher) to conduct peak alignment, peak selection, and quantitation for each metabolite. Metabolites annotation was resorted to three databases (KEGG, Human Metabolome Database (HMDB) and LIPIDMaps). Principal component analysis (PCA) and partial least squares discriminant analysis (PLS-DA) were performed with metaX. Univariate analysis (t test) was used to identify statistical significance (*p* values). Metabolites with variable importance in projection (VIP) > 1, a fold change (FC) > 1.2 or FC < 0.833, and a *p* value < 0.05 were considered to be differentially abundance metabolites (DAMs). The functions of these metabolites and metabolic pathways were marked in the KEGG database. Enrichment of metabolic pathways in differentially abundant metabolites were picked with with x/n > y/N and *p* < 0.05.

### Proteomic sequencing and analysis

Three trees representing infected and healthy *P. koraiensis* were selected as biological replicates for proteomic sequencing. The samples were first ground with liquid nitrogen, and then the powder was moved to a 5 mL centrifuge tube and sonicated three times on ice via a high-intensity ultrasonic processor (Scientz) in lysis buffer. The same amount of Tris-saturated phenol (pH 8.0) was added. The mixture was further vortexed for 5 min. After centrifugation (4 °C, 10 min, 5000 × g), the upper phenol phase was brought to a new centrifuge tube. The proteins were precipitated through the addition of at least four volumes of ammonium-sulphate-saturated methanol and incubated at -20 °C for at least 6 h. After centrifugation at 4 °C for 10 min, the supernatant was ditched. The remaining precipitate was washed with ice-cold methanol and then with ice-cold acetone thrice. The protein was redissolved in 8 M urea, and the protein concentration was determined via a BCA kit following the manufacturer’s instructions. The sample was trypsinized following the instructions of Jingjie PTM BioLab (Hangzhou) Co., Ltd. Finally, the peptides were desalted via a C18 SPE column.

Afterwards, the peptides were dissolved in the mobile phase A used for liquid chromatography and then separated via the EASY-nLC 1200 ultrahigh-performance liquid system. The whole process was instructed by Jingjie PTM BioLab (Hangzhou) Co. Ltd. The data-dependent scanning (DDA) program was employed for data acquisition. In order to increase the efficiency of the mass spectrometer usage, the automatic gain control (AGC) was set to 5E4, 2.5 E5 ions/s for the signal threshold, 40 ms for the maximum injection time, and 30 s for the dynamic rejection time of the tandem mass spectrometry scanning in case of repeated scanning of precursor ions.

The resulting MS/MS spectra data were searched in a database in Proteome Discoverer 2.2 (PD 2.2, Thermo). The search parameters consisted of a mass tolerance of 10 ppm for precursor ion scans and a mass tolerance of 0.02 Da for product ion scans. A maximum of two miscleavage sites were allowed. With regard to protein identification, proteins with at least one unique peptide were identified at a false discovery rate of less than 1.0% at the peptide and protein levels. The performance metrics included Protein FDR ≤ 0.01, Peptide FDR ≤ 0.01, Peptide Confidence ≥ 99%, and XIC width ≤ 75 ppm. Label-free quantification relied on precursor quantification based on intensity. The protein quantification results were statistically analysed by the Mann–Whitney test. Differentially expressed proteins were defined as those with *P* < 0.05 and FC > 1.5 or < 0.67.

### Nematocidal activity assay

Test reagents included Naringenin (99.3% purity by mass), 4-Hydroxyphenylpyruvic Acid (99.7% purity by mass), and 3-Methyl-2-Oxovaleric Acid (99.8% purity by mass), all purchased from MedChemExpress.

The toxicity of the three reagents to *B. xylophilus* was assessed using the nematode-dipping method. The test reagents were dissolved in dimethyl sulfoxide (DMSO) to prepare a master solution, which was subsequently diluted with sterile water to a final concentration of 100 μg/L. Sterile water and DMSO were used as controls 1 and 2, respectively. The drug solution and nematodes solution were mixed at a predetermined volume ratio and transferred into 1.5 mL centrifuge tubes, with each tube containing approximately 1,500 mixed-age pine nematodes. All tubes were incubated at 25 °C, and after 24 h, the number of surviving and deceased nematodes was observed and counted under a microscope.

Due to the pseudo-death phenomenon in PWN, nematodes were considered alive if their bodies displayed wriggling movements in an “S” shape, spiral shape, or curly shape. Conversely, nematodes were deemed dead if their bodies remained stiff and motionless in a “J” or “C” shape. If the body is stiff and motionless in a “J” or “C” shape, with no luster on the surface, and remains unresponsive to repeated stimulation with insect needles, it is considered a dead nematode.

$$\text{Mortality rate }= (\text{The number of dead nematodes }/\text{ Total number of pine nematodes}) \times 100{\%}.$$

$$\text{Corrected mortality rate }= (\text{Mortality rate }-\text{ Control }2\text{ mortality rate}) / (100 -\text{ Control }2\text{ mortality rate}) \times 100{\%}.$$

For the *B. xylophilus* head swing frequency test, 100 nematodes were transferred to 96-well plates containing 300 μL of the drug solution and incubated at a constant temperature of 25 °C for 24 h. After 24 h, immersed pine nematodes were collected and washed three times with sterile water. A head swing was defined as the movement of the nematode’s head from one side to the other and back again. The number of nematode head swings within 30 s was recorded.

### Quantitative real-time polymerase chain reaction (qRT-PCR) analysis

RT-qPCR analysis was conducted using RNA described above. First-strand cDNA was synthesized from 1 µg RNA with ABScript II cDNA Fist-Strand Synthesis Kit (ABclonal), according to the manufacturer’s instructions. The qRT-PCR assay was conducted with 2X Universal SYBR Green Fast qPCR Mix (ABclonal) using an ABI 7500 real-time PCR system (Applied Biosystems). The *Pk18S* gene served as the endogenous control for all qRT-PCR analyses. All samples were independently subjected to three replicate experiments. The relative expression of genes was calculated by using the 2^−∆∆CT^ method.

### Statistical analysis

All data are presented as the means ± standard deviations. The error bars represent the standard deviations based on three independent biological replicates with three technical replicates each.

Significant differences between groups were calculated via one-way ANOVA with Duncan’s range test via SPSS 20.0. *P* < 0.05 was considered to indicate a significant difference.

## Results

### Changes in *P. koraiensis* after *B. xylophilus* inoculation

**Fig. 1:**
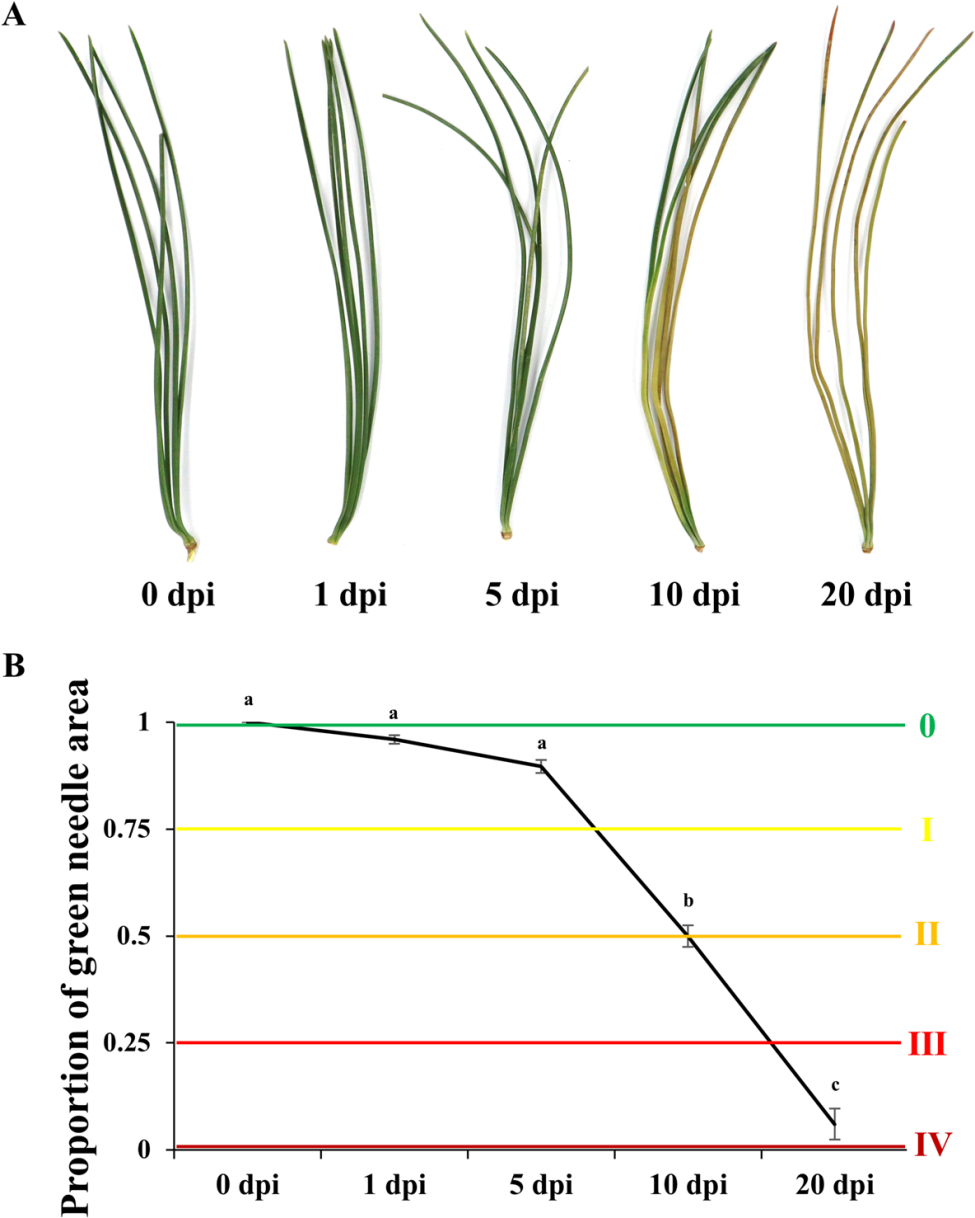
Changes in *P. koraiensis* inoculated with *Bursaphelenchus xylophilus*. **A** Symptoms of *P. koraiensis* after inoculation. **B** Changes in the colour of pine needles. 0: healthy; I: a quarter of the needles were chlorotic; II: half of the needles were chlorotic, turned brown and died, and the branch tip was deformed and bent; III: three-quarters of the needles were chlorotic, turned brown and died, and the branch tip sagged; IV: the needles were chlorotic, turned brown and died, and the whole plant wilted. The error bars indicate the standard deviation. The different letters indicate significant differences (*P* < 0.05)

The symptoms of disease in the inoculated *P. koraiensis* were needle discolouration and wilting (Fig. 1). There were no obvious symptoms at the early stage, and sympotoms showed up at 5 dpi. At 10 dpi, approximately half inoculated pines needles were chlorotic. After 20 days post inoculation, the mortality rate of *P. koraiensis* was greater than 90%. These results suggest that 5 days after PWN infection is the early stage of infection. In addition, we also observed the symptoms of *P. koraiensis* inoculated with ddH_2_O the same time points. These pines remained completely healthy regardless of the inoculation time point (Fig. S1).

To explore the early molecular response of *P. koraiensis* against *B. xylophilus*, we conducted comparative transcriptomic, metabolomic and proteomic analyses between *P. koraiensis* inoculated with PWNs for 5 days (infected) and those inoculated 0 days (healthy).

### Transcriptomic data for infected and healthy *P. koraiensis*

To reveal changes in gene expression levels, transcriptomic analysis was carried out for infected and healthy *P. koraiensis*. We obtained a total of 257,830,864 clean reads, with an average of approximately 42,971,810 clean reads in each sample, ranging from 40,286,922 to 44,224,826 clean reads (Tab. S1). De novo transcriptome assembly with Trinity via reads generated 64,307 unigenes and 106,019 transcripts (Tab. S2). The average length of the unigenes or transcripts was approximately 1 kb (Tab. S2). In addition, more than 85% of the reads in each sample were mapped to the assembled unigenes, suggesting the high abundance and excellent quality of the sequencing data (Tab. S1).

Principal component analysis (PCA) of the read counts of infected and healthy *P. koraiensis* showed an obvious separation between the two types of samples and the close grouping of biological replicates (Fig. S2). Furthermore, the distributions of gene expression values were comparable among all samples (Fig. S2). Differentially expressed gene (DEGs) analysis revealed that 1574 predicted genes were significantly up-regulated and 702 predicted genes were significantly down-regulated in infected *P. koraiensis* compared with healthy *P. koraiensis* (Fig. S2).

### Functional analysis of differentially expressed genes

**Fig. 2:**
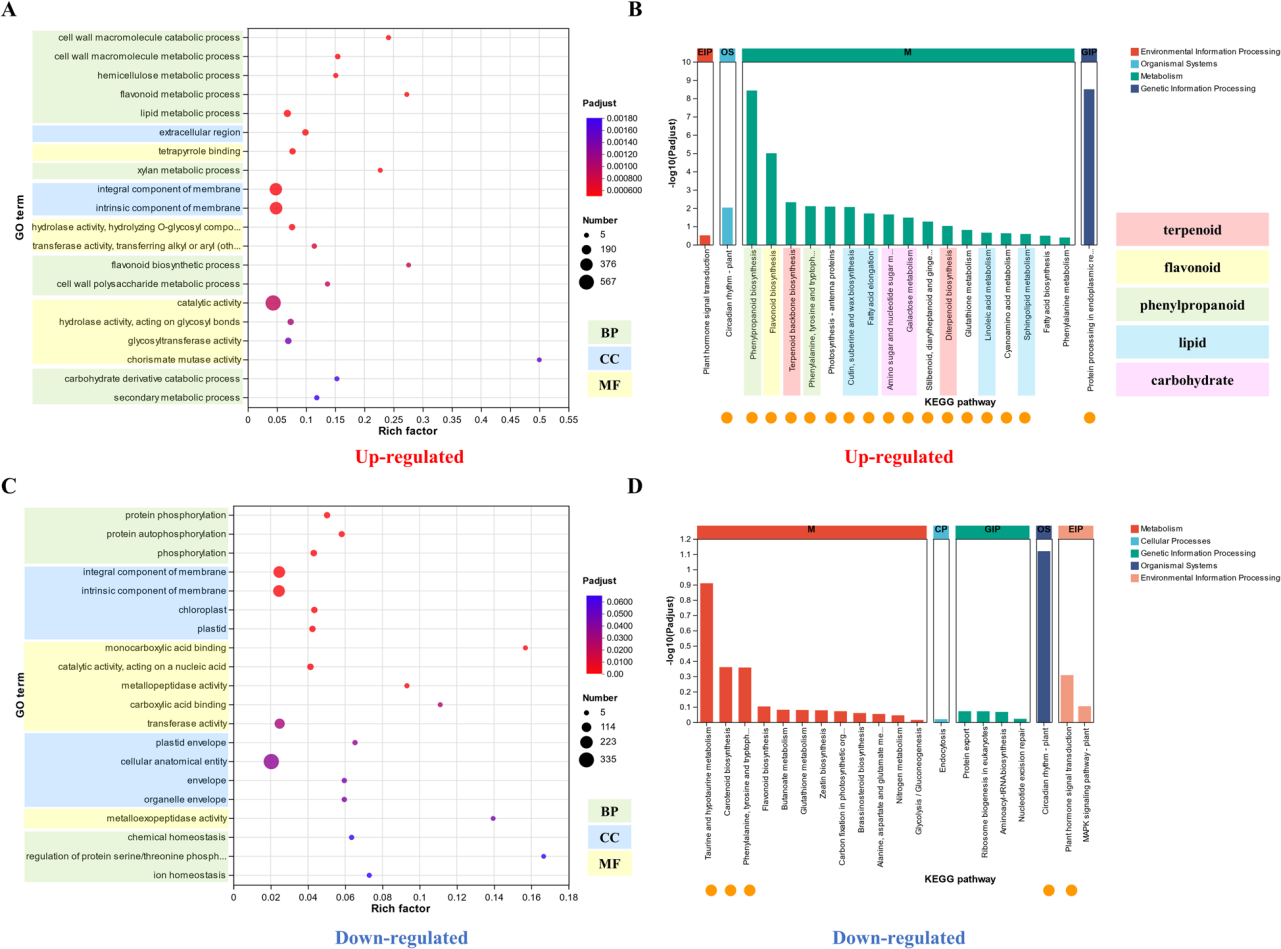
Transcriptome analysis of infected and healthy *P. koraiensis*. **A** Top 20 terms enriched in significantly up-regulated genes in infected *P. koraiensis* compared with healthy *P. koraiensis*. **B** The top 20 enriched KEGG pathways of the genes whose expression was significantly up-regulated in infected *P. koraiensis* compared with healthy *P. koraiensis*. The orange dots represent significant KEGG enrichment. **C** Top 20 terms enriched in genes significantly down-regulated in infected *P. koraiensis* compared with healthy *P. koraiensis*. **D** The top 20 enriched KEGG pathways of the genes whose expression was significantly down-regulated in infected *P. koraiensis* compared with healthy *P. koraiensis*. The orange dots represent the significantly enriched KEGG pathways. The green, blue and yellow boxes represent the BP, CC and MF GO terms, respectively, in (**A**) and (**B**)

GO enrichment analysis of the significantly up-regulated DEGs in infected *P. koraiensis* showed 86 terms significantly enriched (Tab. S3). The top 20 significantly enriched terms included 10 biological process (BP), 3 cellular component (CC) and 7 molecular function (MF) terms (Fig. 2A). All BP terms were related to metabolic processes, including 5 cell wall macromolecule metabolic process terms, 2 flavonoid metabolic process terms, 1 lipid metabolic process term, 1 carbohydrate derivative catabolic process term and 1 secondary metabolic process term. Additionally, 2 terms related to the cell membrane (integral component of the cell membrane and intrinsic components of the cell membrane) and 1 extracellular region term were significantly enriched in the CC categories. Moreover, the 7 significantly enriched MF terms were related to 1 binding function term (tetrapyrrole binding) and 6 catalytic activity function terms, including hydrolase activity, transferase activity and chorismate mutase activity (Fig. 2A).

KEGG enrichment analysis showed 18 significantly enriched KEGG pathways among the significantly up-regulated DEGs of infected *P. koraiensis*, including 15 metabolism pathways, 1 genetic information processing pathway and 1 organismal system pathway (Fig. 2B; Tab. S3). Importantly, 2 terpenoid and polyketide metabolism pathways (terpenoid backbone biosynthesis and diterpenoid biosynthesis), 3 other secondary metabolite biosynthesis pathways (phenylpropanoid biosynthesis, flavonoid biosynthesis and stilbenoid, diarylheptanoid and gingerol biosynthesis), 4 lipid metabolism pathways (cutin, suberine and wax biosynthesis, fatty acid elongation, linoleic acid metabolism and sphingolipid metabolism), 1 amino acid metabolism pathway (alanine, tyrosine and tryptophan biosynthesis) and 2 carbohydrate metabolism pathways (amino sugar and nucleotide sugar metabolism and galactose metabolism) were significantly enriched among the metabolism pathways (Fig. 2B).

Among the genes significantly down-regulated in infected *P. koraiensis*, 17 GO terms were significantly enriched, including 3 BP, 8 CC and 6 MF terms. The 3 significantly enriched BP terms were linked to phosphorylation. The integral component of the cell membrane term and monocarboxylic acid binding term were the terms dominantly enriched in the CC and MF categories respectively (Fig. 2C; Tab S3). KEGG enrichment analysis revealed 5 significantly enriched KEGG pathways among the significantly down-regulated DGEs of infected *P. koraiensis*, which included plant circadian rhythm; taurine and hypotaurine metabolism; carotenoid biosynthesis; phenylalanine, tyrosine and tryptophan biosynthesis; and plant hormone signal transduction (Fig. 2D; Tab S3).

### Key genes involved in the response of *P. koraiensis *to *B. xylophilus* infection

**Fig. 3:**
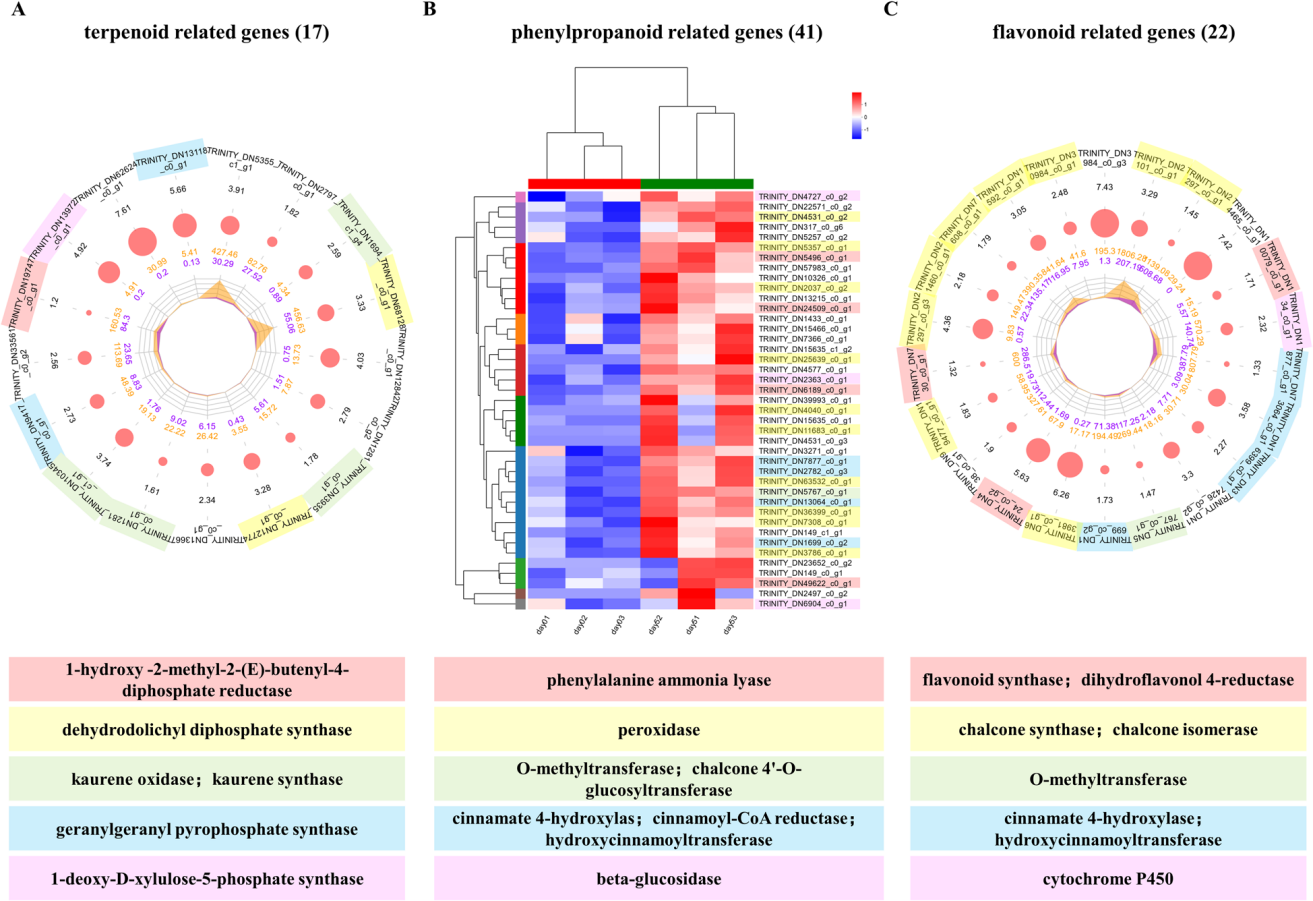
Terpenoid-related genes, phenylpropanoid related-genes and flavonoid-related genes presented significantly increased expression levels in infected *P. koraiensis* relative to healthy plants. **A** The radar chart shows the expression profiles of terpenoid-related genes whose expression was up-regulated in infected *P. koraiensis* compared with healthy plants. The terpenoid gene IDs are displayed in the outermost circle. The penultimate circle of numbers represents the log2(fold change), which is visualized by the red circle. The orange and violet areas in the circle indicate the FPKM of terpenoid-related genes in infected *P. koraiensis* and healthy *P. koraiensis*, respectively. **B** Heatmap showing the expression profiles of phenylpropanoid-related genes in infected and healthy *P. koraiensis*. **C** Radar chart showing the expression profiles of flavonoid-related genes whose expression was up-regulated in infected *P. koraiensis* compared with the healthy plants. The flavonoid-related gene IDs are displayed in the outermost circle. The penultimate circle of numbers represents the log2(fold change), which is visualized by the red circle. The orange and violet areas in the circle indicate the FPKM of flavonoid-related genes in infected *P. koraiensis* and healthy *P. koraiensis*, respectively. The highlighted genes were chalcone-related genes

Numerous resistance-related genes exhibited significantly increased expression levels in infected *P. koraiensis* compared to healthy plants. For example, 19 terpenoid-related genes were significantly up-regulated after infection (Fig. 3A and Tab.S4). Terpenoids are essential players in the interactions and defence reactions between plants, microorganisms, and animals [43]. Among them, 2 genes were annotated as 1-hydroxy-2-methyl-2-(E)-butenyl-4-diphosphate reductase and 1-deoxy-D-xylulose-5-phosphate synthase (DXS). In addition, 2 genes were related to dehydrodolichyl diphosphate synthase (dedol-PP), and 4 genes were associated with kaurene. Moreover, 2 genes were associated with geranylgeranyl pyrophosphate synthase (Fig. 3A and Tab.S4).

Phenylpropanoids and flavonoids are specialized metabolites consistently reported to be involved in plant defence against biotic or abiotic stresses [44]. Our data revealed that 41 genes associated with phenylpropanoids and 22 genes associated with flavonoids were significantly up-regulated in infected *P. koraiensis* compared with healthy plants (Fig. 3B; C and Tab. S5; S6). There were 4 phenylalanine ammonia lyase (PAL) genes and 10 peroxidase genes associated with phenylpropanoids. In addition, 4 genes were associated with cinnamate (including 2 cinnamate 4-hydroxylase: C4H), and 3 genes were associated with beta-glucosidase (Fig. 3B and Tab. S5). Importantly, almost half of the flavonoid-related genes were annotated as chalcones, including 7 chalcone synthase genes and 2 chalcone isomerase genes (Fig. 3C and Tab. S6). In addition, there were 4 genes related to cinnamate, and 1 gene was annotated as a cytochrome P450. Moreover, 2 genes were annotated as dihydroflavonol 4-reductase (DFR), and 1 gene was annotated as a flavonoid synthase (Fig. 3C and Tab. S6).

**Fig. 4:**
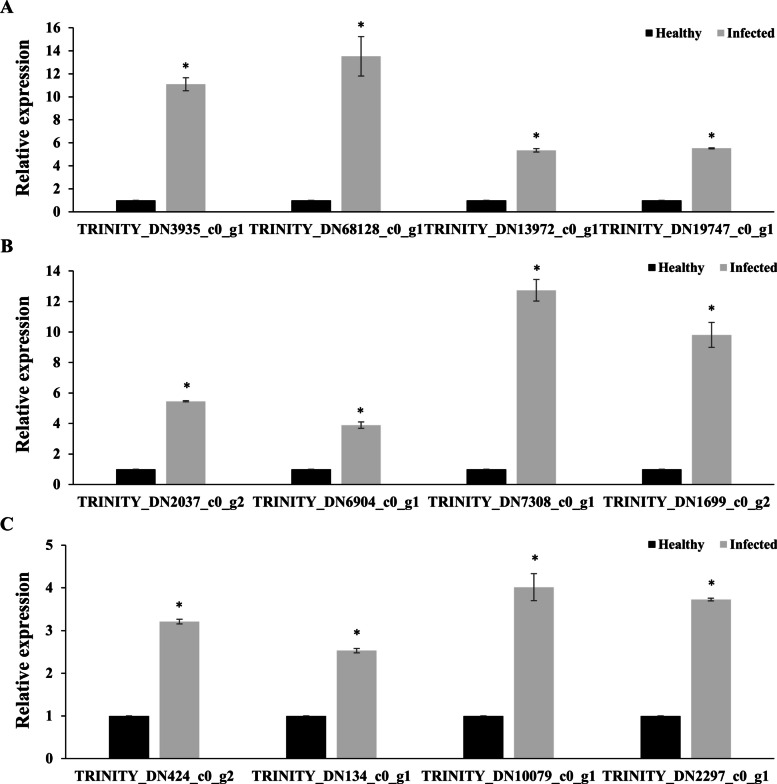
The qRT-PCR confirmation of RNA-Seq results. The qRT-PCR analysis was conducted and the expression levels of four terpenoid related genes (*TRINITY_DN3935_c0_g1*, *TRINITY_DN68128_c0_g1*, *TRINITY_DN13972_c0_g1*, and *TRINITY_DN19747_c0_g1*), four phenylpropanoid related genes (*TRINITY_DN2037_c0_g2*, *TRINITY_DN6904_c0_g1*, *TRINITY_DN7308_c0_g1* and *TRINITY_DN1699_c0_g2*) and four flavonoid related genes (*TRINITY_DN424_c0_g2*, *TRINITY_DN134_c0_g1*, *TRINITY_DN10079_c0_g1* and *TRINITY_DN2297_c0_g1*) were significantly up-regulated in the infected *P. koraiensis* compared with the healthy *P. koraiensis*

To confirm the validity of the transcriptomic data, we performed qRT-PCR analysis of some DEGs described above, including four terpenoid related genes (*TRINITY_DN3935_c0_g1*, *TRINITY_DN68128_c0_g1*, *TRINITY_DN13972_c0_g1*, and *TRINITY_DN19747_c0_g1*), four phenylpropanoid related genes (*TRINITY_DN2037_c0_g2*, *TRINITY_DN6904_c0_g1*, *TRINITY_DN7308_c0_g1* and *TRINITY_DN1699_c0_g2*) and four flavonoid related genes (*TRINITY_DN424_c0_g2*, *TRINITY_DN134_c0_g1*, *TRINITY_DN10079_c0_g1* and *TRINITY_DN2297_c0_g1*). The expression levels of these genes were significantly up-regulated in the infected *P. koraiensis* compared with the healthy *P. koraiensis* (Fig. 4). Consequently, the expression patterns of these genes in the qRT-PCR analysis were basically consistent those detected in the RNA-sequencing analysis, indicating that the transcriptome data were accurate and reliable. All the primers used were listed in the Tab. S7.

### Overview of the metabolomic data of infected and healthy *P. koraiensis*

To clarify the differences in metabolites after *B. xylophilus* infection, samples from infected and healthy *P. koraiensis* were extracted and analysed via mass spectrometry-based metabolomics. Clear separation between the healthy *P. koraiensis* group and the infected *P. koraiensis* group was validated via PCA, which included positive polarity mode and negative polarity mode (Fig. S3). Therefore, the metabolites of healthy *P. koraiensis* and infected *P. koraiensis* were significantly different. In positive polarity mode, the abundances of 217 metabolites were significantly altered, including 36 metabolites whose abundance significantly increased and 181 metabolites, whose abundance significantly decreased. Thirty metabolites were significantly up-regulated, and 141 metabolites were significantly down-regulated, resulting in a total of 174 metabolites in negative polarity mode (Fig. S3).

**Fig. 5:**
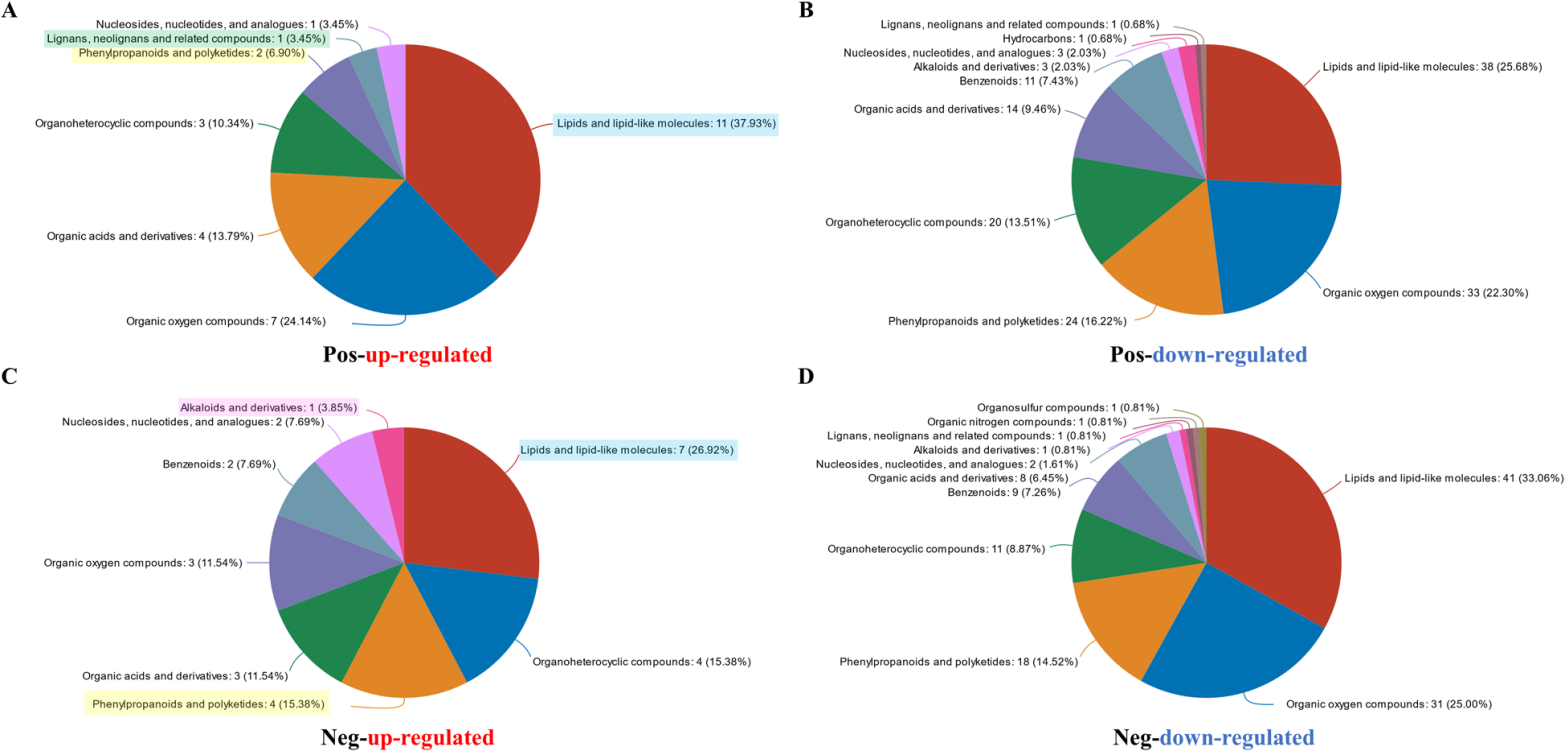
HMDB annotation data of infected and healthy *P. koraiensis*. **A** Up-regulated metabolite categories in positive polarity mode. **B** Down-regulated metabolite categories in positive polarity mode. **C** Up-regulated metabolite categories in negative polarity mode. **D** Down-regulated metabolite categories in negative polarity mode

According to the Human Metabolome Database (HMDB), the up-regulated metabolites were classified into 7 classes, and the down-regulated metabolites were categorized into 10 classes in positive polarity mode. The up-regulated metabolites were classified into 8 classes in negative polarity mode, and the down-regulated metabolites were categorized into 11 classes (Fig. 5A, B, C and D). Importantly, 4 up-regulated metabolites were annotated as plant resistant-related classes. Among them, lignans, neolignans and related compounds only detected in positive polarity mode, and alkaloids and derivatives only detected in negative polarity mode. The other two classes (phenylpropanoids and polyketides; lipids and lipid-like molecules) were detected in both positive and negative polarity mode (Fig. 5A and C).

### Functional analysis of differentially abundant metabolites

**Fig. 6:**
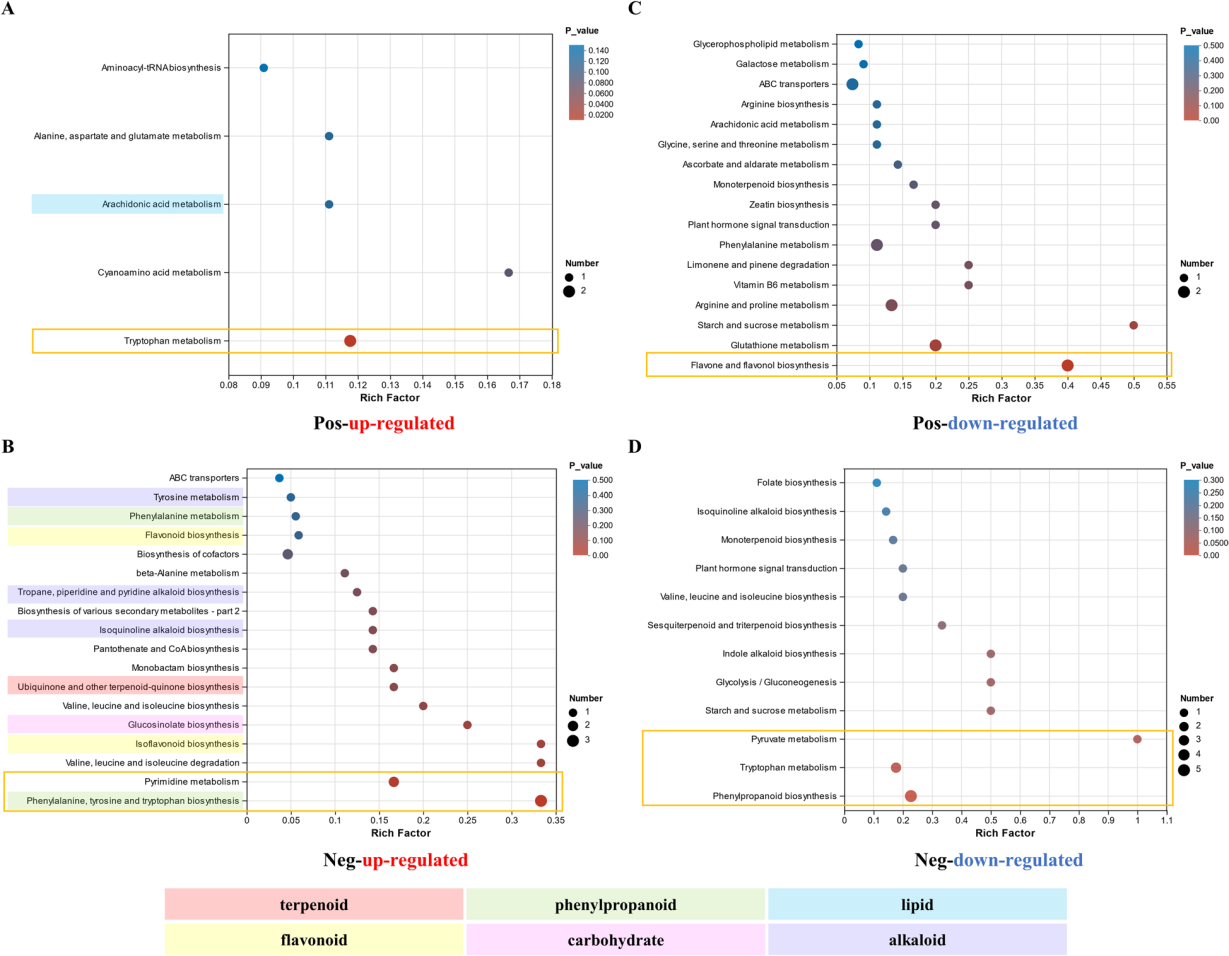
KEGG enrichment analysis of the metabolomic data of infected and healthy *P. koraiensis*. **A** KEGG enrichment of significantly up-regulated metabolites in infected *P. koraiensis* compared with healthy *P. koraiensis* in positive polarity mode. **B** KEGG enrichment of significantly up-regulated metabolites in infected *P. koraiensis* compared with healthy *P. koraiensis* in negative polarity mode. **C** KEGG enrichment of significantly down-regulated metabolites in infected *P. koraiensis* compared with healthy *P. koraiensis* in positive polarity mode. **D** KEGG enrichment of significantly down-regulated metabolites in infected *P. koraiensis* compared with healthy *P. koraiensis* in negative polarity mode, The different shadows represent the various key KEGG pathways. The orange boxes indicate the significantly enriched KEGG pathways

Metabolic KEGG pathway enrichment analyses revealed that 23 pathways were enriched in significantly up-regulated metabolites (Fig. 6A and B). In positive polarity mode, 5 pathways were enriched in significantly up-regulated metabolites, with tryptophan metabolism being one of the significantly enriched pathways. In addition, the enriched arachidonic acid metabolism pathway was related to lipid metabolism (Fig. 6A and Tab. S8).

Among the metabolites that were significantly up-regulated in negative polarity mode, 18 pathways were enriched (Fig. 6B and Tab. S8), of which, 2 pathways were significantly enriched: pyrimidine metabolism and pathways related to phenylalanine metabolism: phenylalanine, tyrosine and tryptophan biosynthesis. Another enriched pathway related to phenylalanine metabolism was phenylalanine metabolism. Moreover, 1 terpenoid and polyketide metabolism pathway was enriched, specifically ubiquinone and other terpenoid-quinone biosynthesis. In addition, 2 pathways, isoflavonoid biosynthesis and flavonoid biosynthesis, were enriched and associated with flavonoid metabolism. Furthermore, 3 enriched pathways were associated with alkaloid biosynthesis: isoquinoline alkaloid biosynthesis; tropane, piperidine and pyridine alkaloid biosynthesis; and tyrosine metabolism. Furthermore, the enriched glucosinolate biosynthesis pathway was a carbohydrate metabolism pathway (Fig. 6B and Tab. S8).

KEGG enrichment analysis revealed 33 enriched pathways among significantly down-regulated metabolites, with 21 pathways in positive polarity mode and 12 pathways in negative polarity mode (Fig. 6C; D and Tab. S8). In positive polarity mode, flavone and flavonol biosynthesis was the pathway significantly enriched, whereas in negative polarity mode, three pathways (phenylpropanoid biosynthesis, tryptophan metabolism and pyruvate metabolism) were significantly enriched (Fig. 6C; D and Tab. S8).

### Key metabolites involved in the response of *P. koraiensis *to *B. xylophilus* infection

**Fig. 7:**
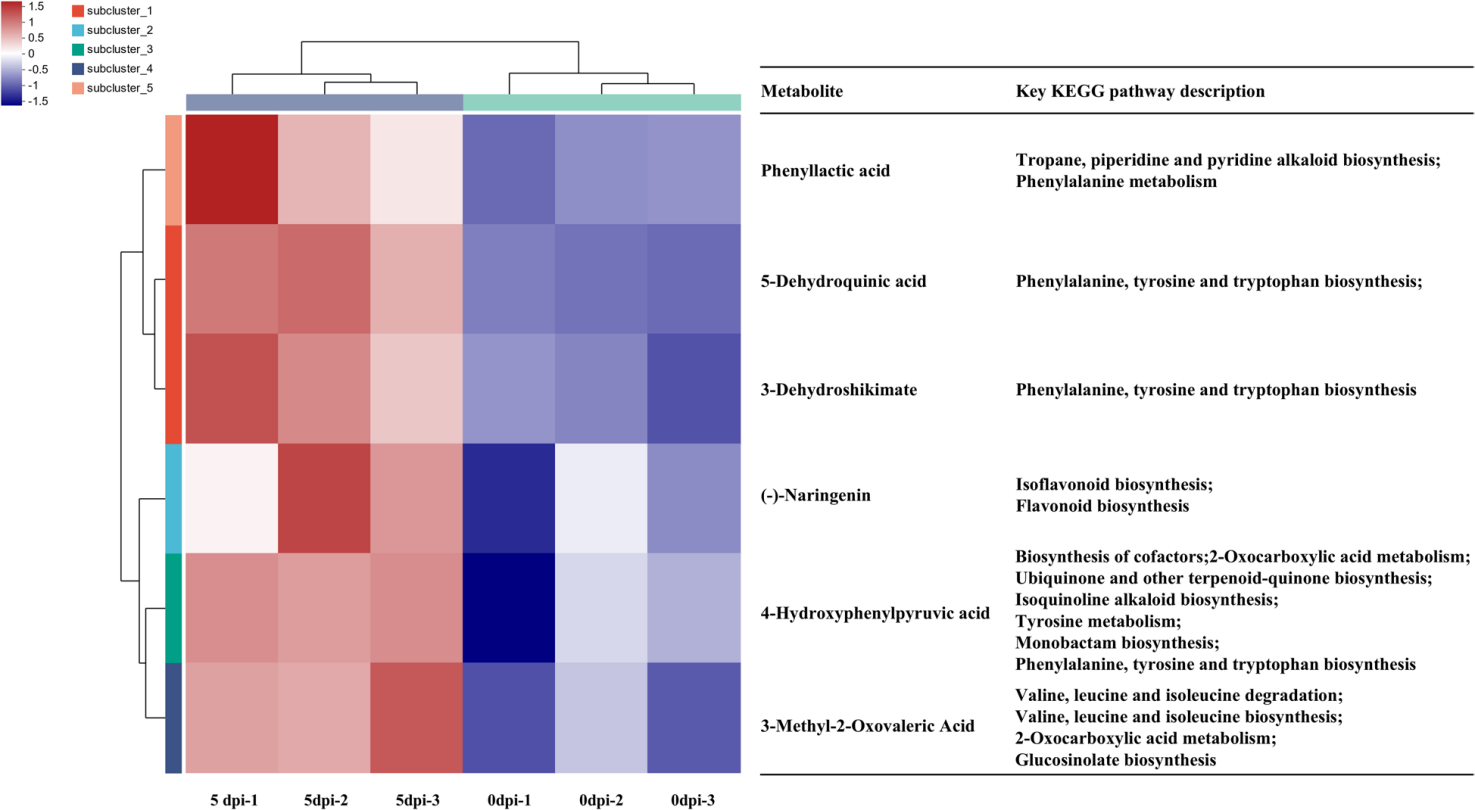
Abundances of key significantly increased abundance metabolites in infected *P. koraiensis* compared with healthy plants

Many resistance-related metabolites showed significantly increased abundance levels in infected *P. koraiensis* relative to healthy plants (Fig. 7 and Tab. S9). For example, phenyllactic acid is involved in tropane, piperidine and pyridine alkaloid biosynthesis and phenylalanine metabolism. In addition, naringenin participates in isoflavonoid biosynthesis and flavonoid biosynthesis. Furthermore, 5-dehydroquinic acid and 3-dehydroshikimate participate in phenylalanine, tyrosine and tryptophan biosynthesis. Moreover, 4-hydroxyphenylpyruvic acid (HPPA) is associated with many important pathways: biosynthesis of cofactors, 2-oxocarboxylic acid metabolism; ubiquinone and other terpenoid-quinone biosynthesis; isoquinoline alkaloid biosynthesis; tyrosine metabolism; monobactam biosynthesis; and phenylalanine, tyrosine and tryptophan biosynthesis. 3-Methyl-2-oxovaleric acid is also involved in several important pathways: valine, leucine and isoleucine degradation and biosynthesis; 2-oxocarboxylic acid metabolism; and glucosinolate biosynthesis (Fig. 7 and Tab. S9).

**Fig. 8:**
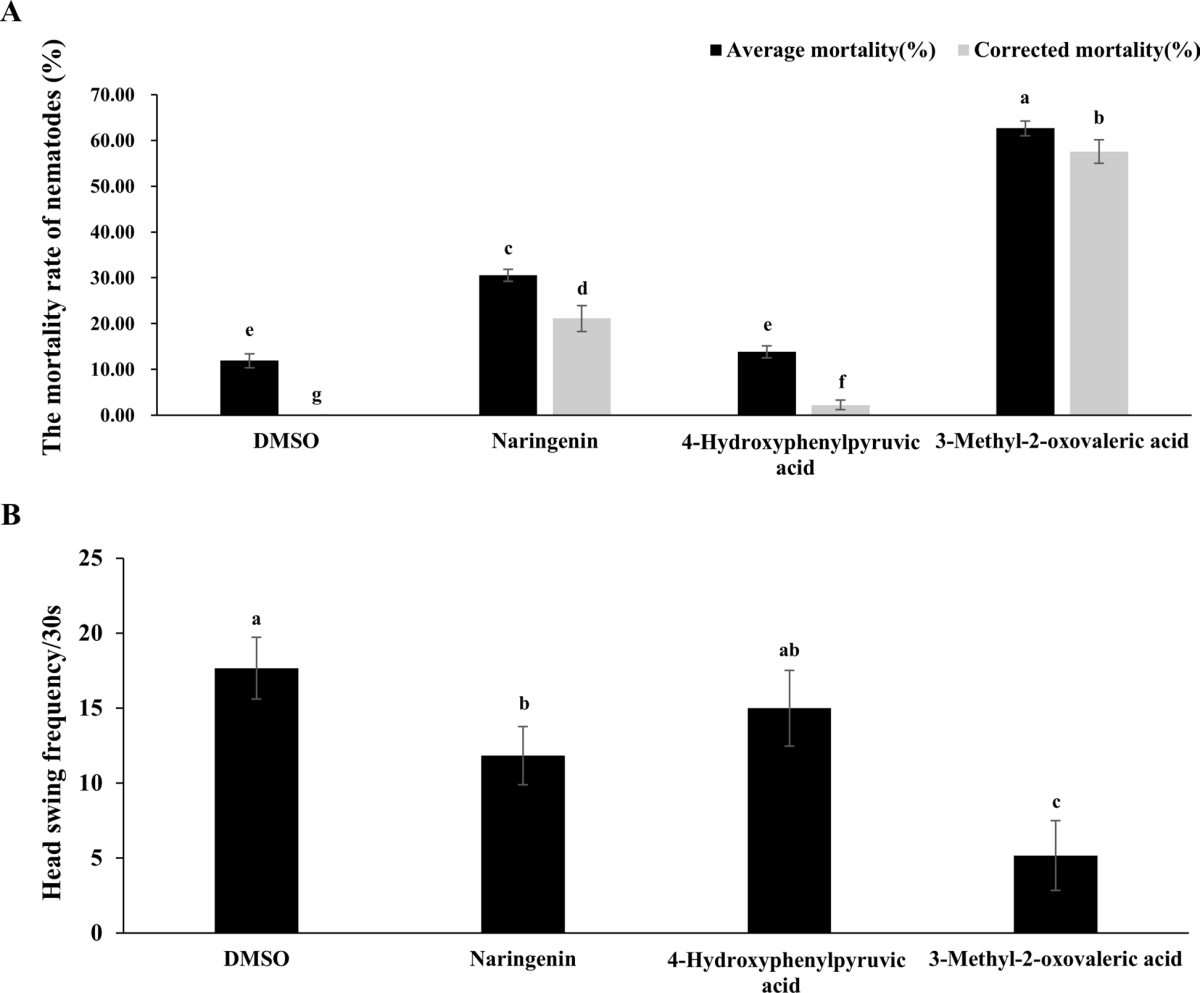
The mortality rate of nematodes and the head swing frequency of nematodes treated with three selected metabolites

The mortality rate of nematodes and the head swing frequency of nematodes treated with three selected metabolites (Naringenin, 4-Hydroxyphenylpyruvic acid and 3-Methyl-2-Oxovaleric Acid) were analyzed. Among them, two metabolites significantly increased the mortality rate of *B. xylophilus* compared to the DMSO control. Naringenin demonstrated a toxic effect on *B. xylophilus*, with an average mortality rate of 30.54% and a significantly elevated corrected mortality rate of 21.14%. Furthermore, 3-Methyl-2-oxovaleric acid exhibited a highly significant toxic effect, with an average mortality rate of 62.65% and a corrected mortality rate of 57.59%. In contrast, 4-Hydroxyphenylpyruvic acid exhibited a mortality rate similar to that of the DMSO control, indicating minimal toxicity (Fig. 8A).

Head swing, a biomarker of muscle activity, was used to investigate the moving behavior of nematodes. To examine whether three selected metabolites affect the moving of *B. xylophilus*, the frequency of head swing was measured. No significant change in head swing frequency was observed following immersion in 4-Hydroxyphenylpyruvic acid compared to the DMSO control. However, under 3-Methyl-2-oxovaleric acid treatment, the head swing frequency of *B. xylophilus* was about 5 times in 30 s, which was significantly reduced compared with the control DMSO. Similarly, exposure to Naringenin also caused a significant reduction in head swing frequency, with approximately 12 times in 30 s. These results indicating that 3-Methyl-2-oxovaleric acid and Naringenin threatened the movement of *B. xylophilus* (Fig. 8B).

### Proteome analysis between infected and healthy *P. koraiensis*

**Fig. 9:**
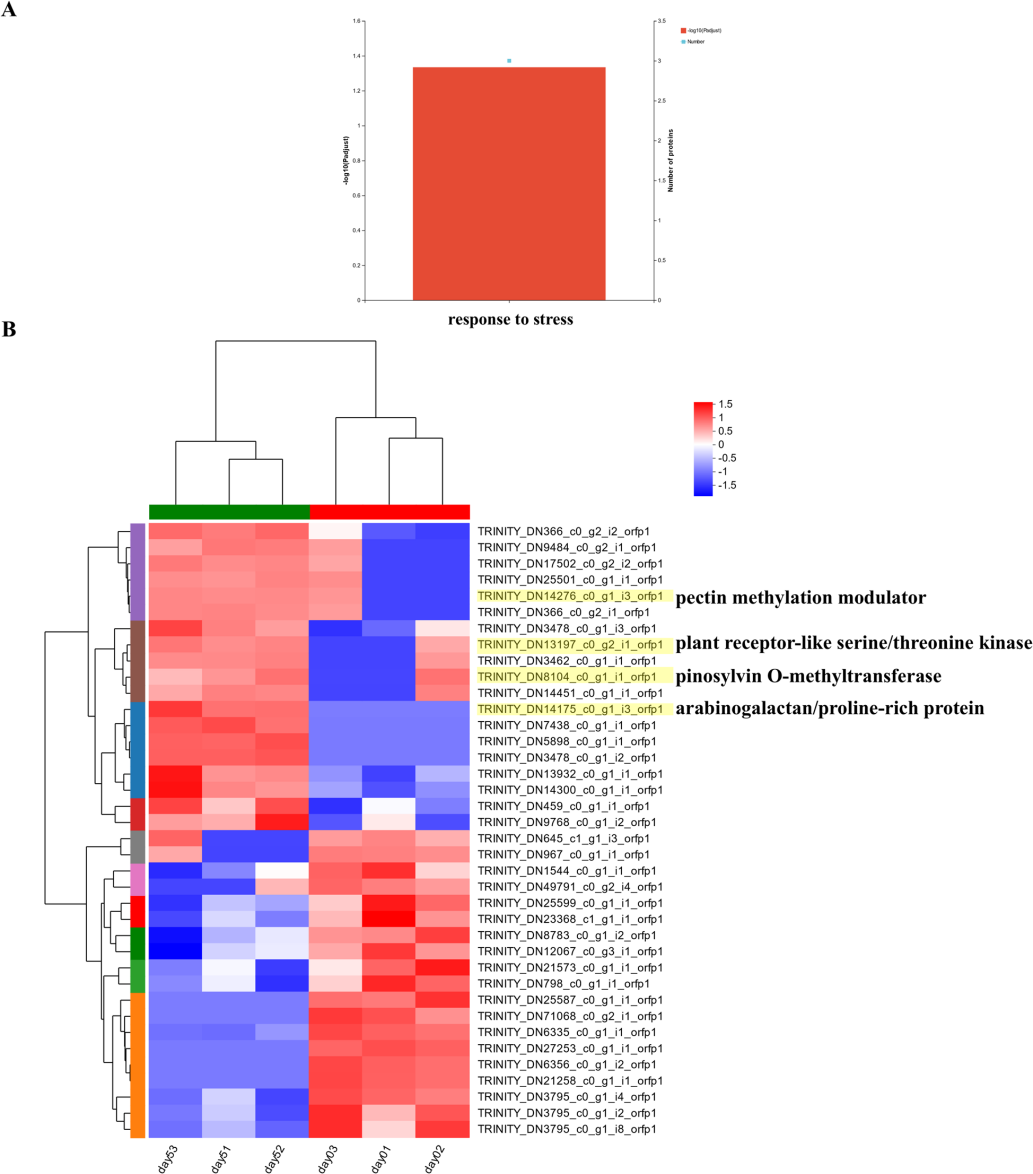
Proteome analysis of infected and healthy *P. koraiensis*. **A** GO significant enrichment of significantly up-regulated proteins in infected *P. koraiensis* compared with healthy *P. koraiensis*. **B** Heatmap showing the expression levels of proteins differentially abundant in infected and healthy *P. koraiensis*

To determine sample changes in protein levels, the abundance of proteins was calculated and normalized based on all detected peptides. The PCA results based on the proteomic data showed clear separation between infected and healthy *P. koraiensis* samples, demonstrating the reliability of the sampling and results (Fig. S4A). The abundance of 38 proteins significantly differed between infected *P. koraiensi* and healthy plants, suggesting 19 proteins whose abundance significantly increased and 19 proteins whose abundance significantly decreased (Fig. S4B). In addition, 1 GO term related to the response to stress and belonging to the BP category, was significantly enriched among up-regulated proteins (Fig. 9A).

Among the 19 proteins whose abundance increased, 6 proteins were specifically detected in infected *P. koraiensis* but not in healthy plants. Among them, 4 proteins were annotated. DN8104 and DN14175 were associated with pinosylvin O-methyltransferase and arabinogalactan/proline-rich proteins, respectively. Additionally, DN13197 was annotated as a plant receptor-like serine/threonine kinase. Furthermore, DN14276 was related to pectin methylation modulators (Fig. 9B).

### Response network of *P. koraiensis* in the early defence against *B. xylophilus* infection

**Fig. 10:**
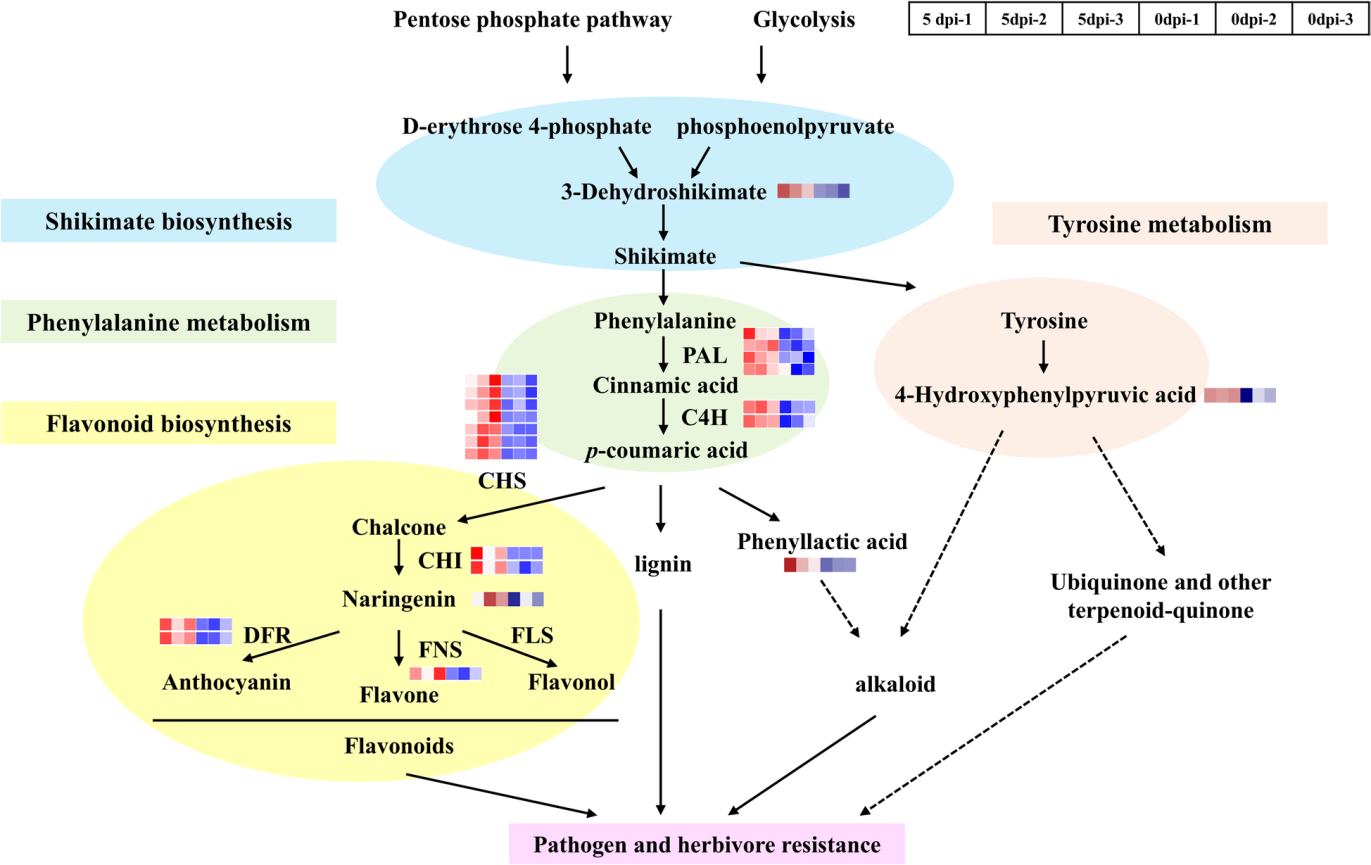
Scheme of alterations in *P. koraiensis* after *B. xylophilus* infection. Abbreviations indicate enzymes. PAL: phenylalanine ammonia lyase; C4H: cinnamic acid 4‐hydroxylase; CHS: chalcone synthase; CHI: chalcone isomerase; FNS: flavone synthase; FLS: flavonol synthase; DFR: dihydroflavonol 4-reductase. The heatmaps showed the corresponding metabolite abundances and gene expression levels alongside the relevant substances and genes

Integrated transcriptomic, metabolomic and proteomic analyses revealed that *P. koraiensis* could actively respond to *B. xylophilus* via various defence strategies (Fig. 10). According to the metabolomic data, the abundance of 3-dehydroshikimate, a metabolite in the shikimate pathway, significantly increased (Fig. 7).

Phenylalanine and tyrosine are derived from the final product of the shikimate pathway [45]. According to the metabolomic data, the abundance of 4-hydroxyphenylpyruvic acid (HPPA), which is derived from tyrosine and involved in the biosynthesis of ubiquinone, other terpenoid-quinone, and isoquinoline alkaloids, significantly increased (Fig. 7). In addition, genes annotated as phenylalanine ammonia lyase (PAL) and cinnamic acid 4‐hydroxylase (C4H), which catalyse the first two steps of the phenylpropanoid pathway, were significantly up-regulated (Fig. 3). The phenylpropanoid pathway is associated with both core and specialized metabolism and provides precursors for all downstream metabolites [46].

Flavonoid biosynthesis and lignin biosynthesis are important branches of phenylpropanoid metabolism [47]. The abundance of naringenin and the expression of chalcone synthase (CHS) genes and chalcone isomerase (CHI) genes significantly increased in infected plants relative to healthy plants (Figs. 3 and 7). Furthermore, the flavonoid synthase (FNS) and dihydroflavonol 4-reductase (DFR) genes were also significantly up-regulated according to our transcriptomic data (Fig. 3). The metabolomic data revealed many up-regulated metabolites annotated as lignans, neolignans and related compounds as well as alkaloids and derivatives in positive polarity mode and negative polarity mode (Fig. 5A and C). Moreover, phenyllactic acid, which may be involved in alkaloid biosynthesis, was significantly up-regulated (Fig. 7).

## Discussion

In our study, we observed symptoms in *P. koraiensis* after *B. xylophilus* inoculation and conducted comparative transcriptomic, metabolomic and proteomic analyses between inoculated *P. koraiensis* inoculated 5 dpi and 0 dpi. Many genes, including terpenoid-, phenylpropanoid- and flavonoid-related genes, were significantly up-regulated at 5dpi. Similarly, many metabolites, including 3-dehydroshikimate, naringenin, phenyllactic acid, and 4-hydroxyphenylpyruvic acid, significantly increased in abundance. In the functional analysis of significantly up-regulated genes and metabolites, many terms were enriched in terpenoid-, phenylpropanoid-, flavonoid-, carbohydrate- and alkaloid-related pathways. In addition, pinosylvin O-methyltransferase, arabinogalactan/proline-rich proteins, plant receptor-like serine/threonine kinase and pectin methylation modulators were significantly up-regulated according to the proteomic data. Therefore, *P. koraiensis* can actively respond to *B. xylophilus* via various defence strategies.

Our RNA-Seq data revealed that the expression levels of terpenoid-related genes, such as those for dehydrodolichyl diphosphate synthase (dedol-PP), 1-hydroxy-2-methyl-2-(E)-butenyl-4-diphosphate reductase and 1-deoxy-D-xylulose-5-phosphate synthase (DXS) significantly increased in infected *P. koraiensis*. Dedol-PP synthase is a key enzyme involved in the biosynthesis of dolichol, which is a type of isoprenoid that plays essential roles in *Arabidopsis thaliana* growth and development [48]. DXS are the enzymes in the MEP pathway, which is the first major stage of terpenoid biosynthesis [49]. In addition, terpenoid backbone biosynthesis and diterpenoid biosynthesis were significantly enriched among the KEGG pathways associated with significantly up-regulated DEGs in infected *P. koraiensis*. Furthermore, the abundance of 4-hydroxyphenylpyruvic acid was up-regulated according to the metabolomic data, and ubiquinone and other terpenoid-quinone biosynthesis pathways were enriched. Terpenoids are the most abundant secondary metabolites in plants and play a predominant role in a plant’s ability to cope with biotic stress [50, 51]. Terpenoid mixtures may be dimissed as defending barriers for conifers against pests and pathogens like bark beetles, weevils, and their associated fungi [43, 52, 53]. GO enrichment analysis of DEGs in previous work on the transcriptomic response of *P. assoniana* to *B. xylophilus* also revealed “terpenoid biosynthesis” [6, 54]. In addition, terpenoid-related events are involved in the molecular response of *Pinus yunnanensis* to PWN infection [20]. These results are similar to our findings, therefore, terpenoids may serve as a source of *Pinus* defence.

Moreover, pinosylvin, a natural preinfectious stilbenoid toxin, is a terpenoid polyphenol compound principally found in the heartwood of *Pinus* spp. (e.g., *Pinus sylvestris*) and in pine leaves (*Pinus densiflora*) [55]. It provides defence against pathogens and insects for many plants. Pinosylvin acts as a functional compound that is responsible for defence against pathogens and insects in a wide range of plants, especially pines [56]. Our proteomic data revealed that the abundance of pinosylvin O-methyltransferase increased significantly after infection, which may be involved in pinosylvin biosynthesis. In addition, pinosylvin O-methyltransferase, arabinogalactan/proline-rich proteins, plant receptor-like serine/threonine kinase and pectin methylation modulators (DN8104, DN14175, DN13197 and DN14276) were specifically detected in infected *P. koraiensi* but were no abundance in the healthy plants. Thus these proteins could serve as potential targets for PWD detection in the future.

The shikimate pathway connects central carbon metabolism (glycolysis and the pentose phosphate pathways) [45]. Two carbon metabolism pathways, amino sugar and nucleotide sugar metabolism as well as galactose metabolism, were significantly enriched among the significantly up-regulated DEGs of infected *P. koraiensis*. In addition, glucosinolate biosynthesis was enriched in KEGG pathways among the significantly up-regulated metabolites in our data. The shikimate pathway is closely linked to aromatic amino acids (tryptophan, phenylalanine, and tyrosine) and involves numerous aromatic secondary metabolites such as alkaloids, flavonoids, lignins, and aromatic antibiotics [45]. Many of these compounds are bioactive and play important roles in plant defence against biotic and abiotic stressors and environmental interactions [57]. The 3-dehydroshikimate intermediate is efficiently converted into shikimate, therefore increasing metabolic flux through the shikimate pathway [45]. In our study, 3-dehydroshikimate significantly increased in abundance in infected *P. koraiensis*. Thus, it likely serve as a key precursor for a wide range of important secondary metabolites. Tyrosine is a final product of the shikimate pathway and participates in 4-hydroxyphenylpyruvic acid (HPPA) biosynthesis, which significantly increased in abundance in infected *P. koraiensis*. HPPA is associated with the biosynthesis of ubiquinone and other terpenoid-quinone compounds; isoquinoline alkaloid biosynthesis; and phenylalanine, tyrosine and tryptophan biosynthesis [58, 59].

Phenylalanine is another product from the shikimate pathway and leads to the biosynthesis of phenylpropanoid metabolites [60]. Phenylpropanoid biosynthesis can be induced under wounded or stress conditions, and its synthesis is strongly associated with defence responses [61]. In our work, 41 phenylpropanoid-related genes, including phenylalanine ammonia lyase (PAL) and cinnamate 4-hydroxylase (C4H), were significantly up-regulated after infection. The first two steps of the phenylpropanoid pathway are connected to both core and specialized metabolism, providing precursors for all of the downstream metabolites [62]. The reactions are catalysed by PAL and C4H [46]. PAL directs metabolic flux from the shikimate pathway to various branches of phenylpropanoid metabolism by catalyzing the formation of trans‐cinnamic acid from phenylalanine [63]. C4H is a cytochrome P450‐dependent monooxygenase that catalyses the second step of the general phenylpropanoid pathway, namely, the hydroxylation of cinnamic acid to generate *p*‐coumaric acid [63]. Furthermore, phenylpropanoid biosynthesis as well as phenylalanine, tyrosine and tryptophan biosynthesis were significantly enriched in the KEGG pathways in DEGs significantly up-regulated in infected *P. koraiensis*. In addition, 6.9% and 15.38% of the significantly up-regulated metabolites were annotated as phenylpropanoids and polyketides in our data. Similarly, in *Pinus pinaster*, 72 genes associated with the phenylpropanoid pathway were differentially expressed [64]. In other *Pinus pinaster* studies, similar results were acquired, with the induction of several genes involved in the phenylpropanoid pathway, including those involved in flavonoid or lignin biosynthesis [8]. Phenylpropanoid metabolism produces end products such as flavonoids, lignans, and tannins [65].

Flavonoid biosynthesis is a vital branch of phenylpropanoid metabolism and brings about the largest class of polyphenolic metabolites [47, 66]. Chalcone synthase (CHS) is the first enzyme involved in flavonoid biosynthesis; it acts on p‐coumaroyl‐CoA (from *p*‐coumaric acid) as a substrate to synthesize chalcone and directs metabolic flux to flavonoid metabolism [44]. Chalcones can be moved into aurones with variations in the 3′‐position. Chalcone isomerase (CHI) acts on chalcones to generate flavanones such as naringenin [47]. In our study, genes associated with CHS and CHI were significantly up-regulated in infected *P. koraiensis*. In addition, flavonoid biosynthesis was significantly enriched KEGG pathway in the significant up-regulated DEGs in infected *P. koraiensis*. Furthermore, the abundance of naringenin significantly increased after infection. Moreover, naringenin can be converted into flavonols, flavones, and anthocyanins by flavonol synthase (FLS), flavone synthase (FNS) and dihydroflavonol 4-reductase (DFR), which protect plants against pathogens and herbivores [46]. Among the significantly up-regulated DEGs, 2 genes were annotated as DFR, and 1 gene was annotated as a flavonoid synthase in our study. Anthocyanins and flavonols were found to be more abundant on the red side than on the green side of red‐blushed mango fruit, which are efficient at inhibiting *Colletotrichum gloeosporioides* [67]. Flavones function as phytoalexins in plant defence [68]. Moreover, flavonoid-related GO terms and KEGG pathways were enriched according to metabolomic data. These results suggest that flavonoids play an important role in the resistance of *P. koraiensis* to PWN infection. Similar results were also found in *P. massoniana*. In *P. massoniana*, chalcone synthase genes were dominant within the module enriched with genes highly correlated with nematode populations [22].

The lignin pathway is a major branch downstream of the general phenylpropanoid pathway [69]. Lignin is a heterogeneous phenolic polymer stemed from the oxidative polymerization of hydroxycinnamoyl alcohol derivatives and is stored in the secondary cell wall of all vascular plants [70]. Lignin forms a barrier against microbial infections and pest herbivory, acting as one of the major contributors to biotic stress resistance [71, 72]. According to our metabolomic data, 3.45% of the significantly up-regulated metabolites were annotated as lignans, neolignans or related compounds. Our results suggest that lignification is an efficient strategy by which plant slow the sprawl of PWN, which damages the tissue of plant, and is likely to intervene with nematode feeding of plant cells [73, 74].

## Conclusion

Our results revealed *P. koraiensis* can actively respond to *B. xylophilus* via various defence strategies, including terpenoid-, flavonoid-, phenylpropanoid-, alkaloid- and lipid-related events. Particularly, terpenoids and flavonoids are required for the early defence responses of* P. koraiensis* to *B. xylophilus* infection.

## Supplementary Information

Supplementary Material 1: Figure S1. The symptoms of *Pinus koraiensis* inoculated with ddH_2_O at the same time points.

Supplementary Material 2: Figure S2. Gene expression data for infected and healthy *P. koraiensis*. (A) Principal component analysis of the transcriptomic data of infected and healthy* P. koraiensis*. (B) Global view of the distribution of gene expression levels in infected and healthy *P. koraiensis*. (C) Global view of the number of differentially expression genes between infected and healthy *P. koraiensis*.

Supplementary Material 3: Figure S3. Overview of metabolomic data between infected and healthy *P. koraiensis*. (A) Principal component analysis of infected and healthy *P. koraiensis* during LC-MS/MS analysis in positive mode. (B) Principal component analysis of infected and healthy *P. koraiensis* during LC-MS/MS analysis in negative mode. (C) A global view of the number of differentially abundant metabolites between infected and healthy *P. koraiensis* in positive polarity mode. (D) A global view of the number of differentially abundant metabolites between infected and healthy *P. koraiensis* in negative polarity mode.

Supplementary Material 4: Figure S4. Overview of proteomic data of infected and healthy *P. koraiensis*. (A) Principal component analysis of the proteomic data of infected and healthy *P. koraiensis*. (B) Global view of the number of differentially abundant proteins in infected and healthy *P. koraiensis*.

Supplementary Material 5: Table S1. RNA-Seq statistics.

Supplementary Material 6: Table S2. Results of the de novo transcriptome assembly performed with Trinity.

Supplementary Material 7: Table S3. GO significant enrichment data and KEGG significant enrichment data. (Sheet 1) GO enrichment analysis of genes significantly up-regulated between infected and healthy *P. koraiensis*. (Sheet 2) GO enrichment analysis of genes significantly down-regulated between infected and healthy *P. koraiensis*. (Sheet 3) KEGG enrichment analysis of genes significantly up-regulated between infected and healthy* P. koraiensis*. (Sheet 4) KEGG enrichment analysis of genes significantly down-regulated between infected and healthy *P. koraiensis*.

Supplementary Material 8: Table S4. Genes related to terpenoids significantly up-regulated in infected *P. koraiensis* relative to healthy plants.

Supplementary Material 9: Table S5. Genes related to phenylpropanoids significantly up-regulated in infected *P. koraiensis* relative to healthy plants.

Supplementary Material 10: Table S6. Genes related to flavonoids significantly up-regulated in infected *P. koraiensis* relative to healthy plants.

Supplementary Material 11: Table S7. Primers used in this study.

Supplementary Material 12: Table S8. KEGG pathway enrichment between infected and healthy *P. koraiensis* according to the metabolomic data. (Sheet 1) KEGG enrichment data of significantly up-regulated metabolites in infected *P. koraiensis* compared with healthy *P. koraiensis* in positive polarity mode. (Sheet 2) KEGG enrichment data of significantly down-regulated metabolites in infected *P. koraiensis* compared with healthy *P. koraiensis* in positive polarity mode. (Sheet 3) KEGG enrichment data of significantly up-regulated metabolites in infected *P. koraiensis* compared with healthy *P. koraiensis* in negative polarity mode. (Sheet 4) KEGG enrichment data of significantly down-regulated metabolites in infected *P. koraiensis* compared with healthy *P. koraiensis* in negative polarity mode.

Supplementary Material 13: Table S9. Key metabolite data for *P. koraiensis* responses to *B. xylophilus* infection.

## Data Availability

The transcriptome data used in this study were uploaded to the NCBI SRA, with accession: PRJNA1194158 and the accession numbers of raw reads were SRX26974804, SRX26974805, SRX26974806 (Pinus koraiensis inoculated with Bursaphelenchus xylophilus for 5 days 1-3), and SRX26974807, SRX26974808, SRX26974809 (Pinus koraiensis inoculated with Bursaphelenchus xylophilus for 0 days 1-3). The mass spectrometry proteomics data have been deposited to the ProteomeXchange Consortium (https://proteomecentral.proteomexchange.org) via the iProX partner repository with the dataset identifier PXD058632. We have submitted our metabolomics data to MetaboLights with the accession numbers: MTBLS11855 (www.ebi.ac.uk/metabolights/MTBLS11855).
