# Supplementary figures and images for: Transcriptomic, metabonomic and proteomic analyses reveal that terpenoids and flavonoids are required for Pinus koraiensis early defence against Bursaphelenchus xylophilus infection
Source: BMC Plant Biol. 2025 Feb 12;25:185. doi: 10.1186/s12870-025-06192-8 (PMC11816754; doi:10.1186/s12870-025-06192-8)

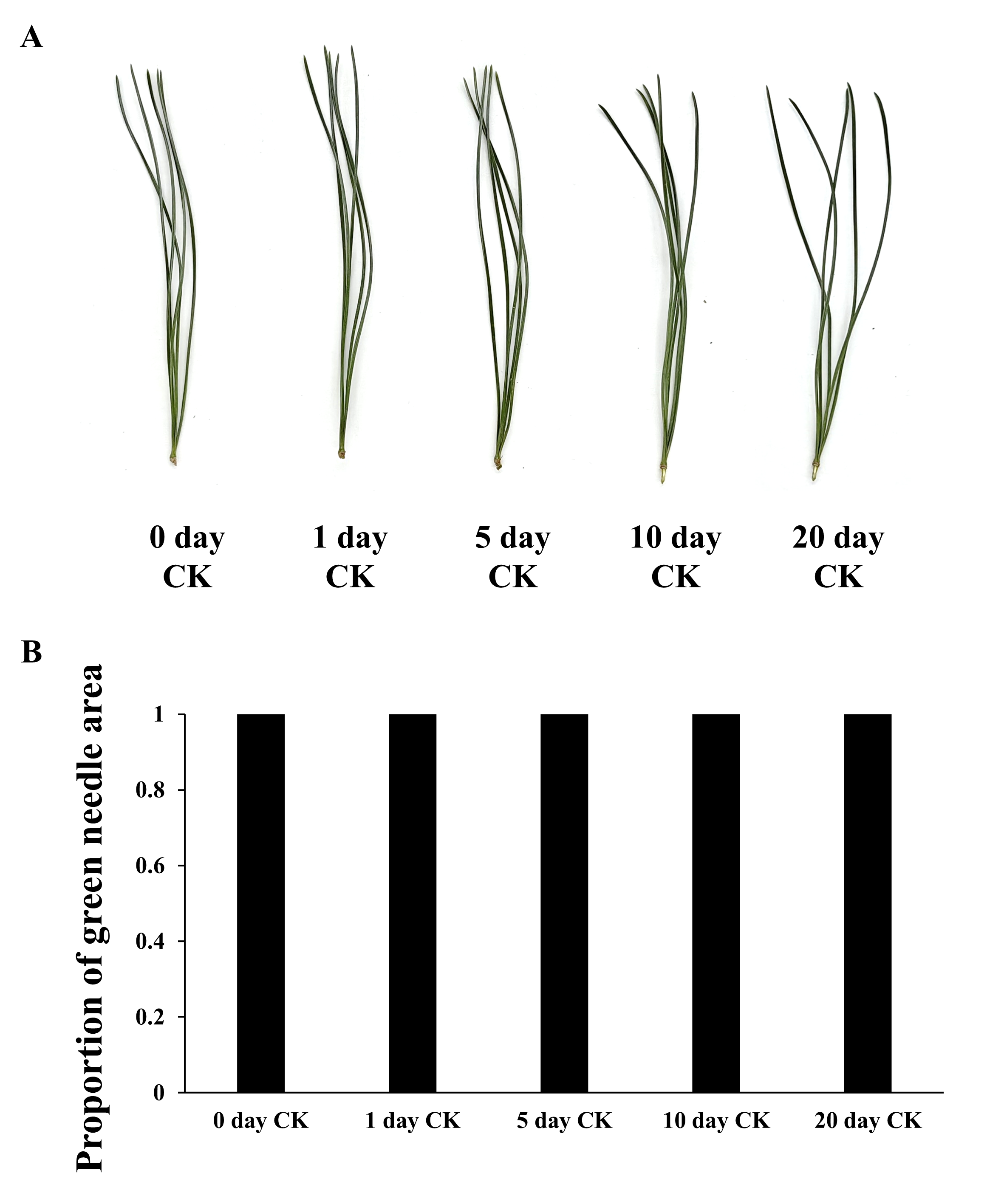

Supplement: Supplementary file 1 — Supplementary Material 1: Figure S1. The symptoms of Pinus koraiensis inoculated with ddH2O at the same time points. [file 12870_2025_6192_MOESM1_ESM.tif]

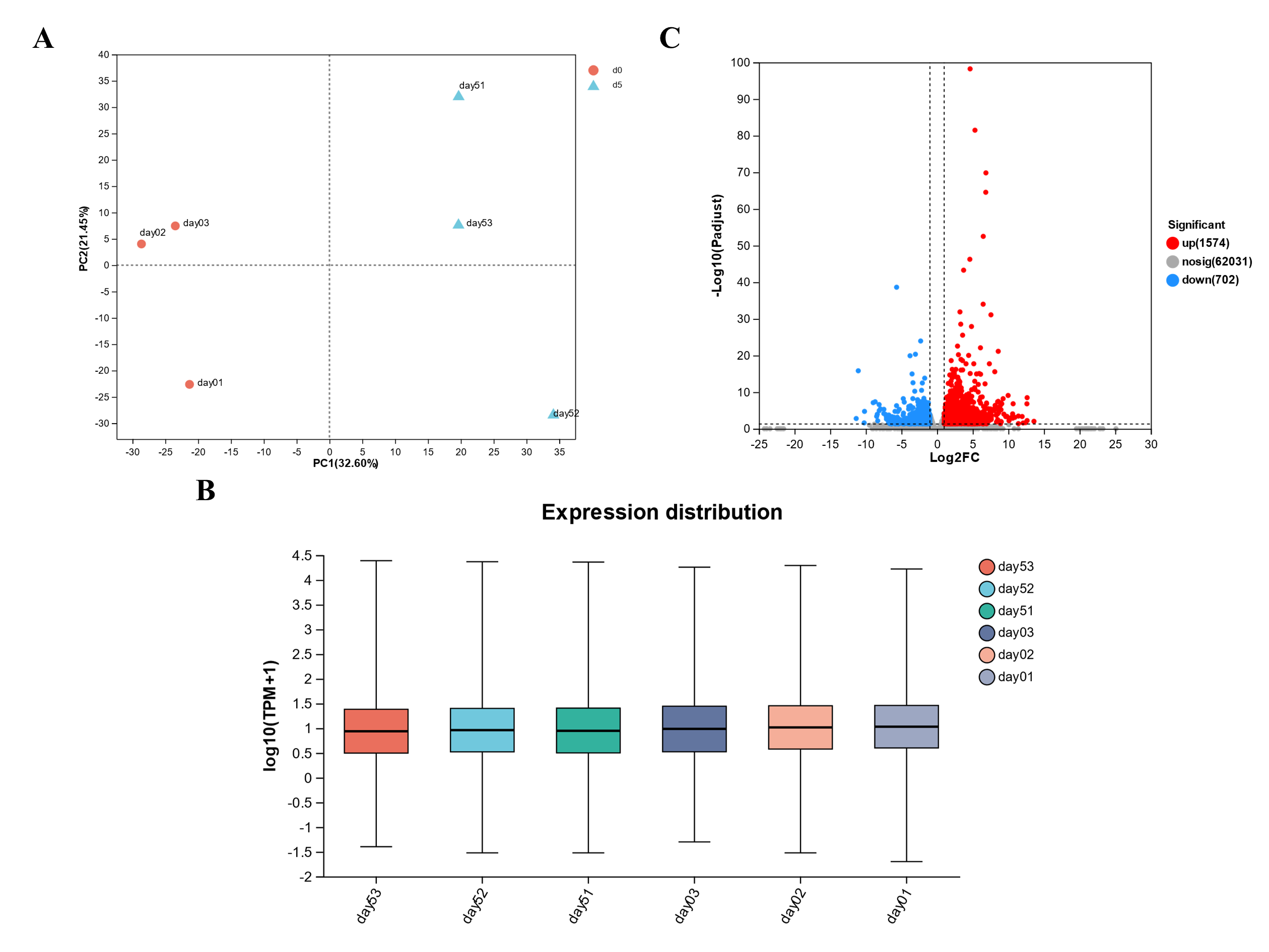

Supplement: Supplementary file 2 — Supplementary Material 2: Figure S2. Gene expression data for infected and healthy P. koraiensis. (A) Principal component analysis of the transcriptomic data of infected and healthy P. koraiensis. (B) Global view of the distribution of gene expression levels in infected and healthy P. koraiensis. (C) Global view of the number of differentially expression genes between infected and healthy P. koraiensis. [file 12870_2025_6192_MOESM2_ESM.tif]

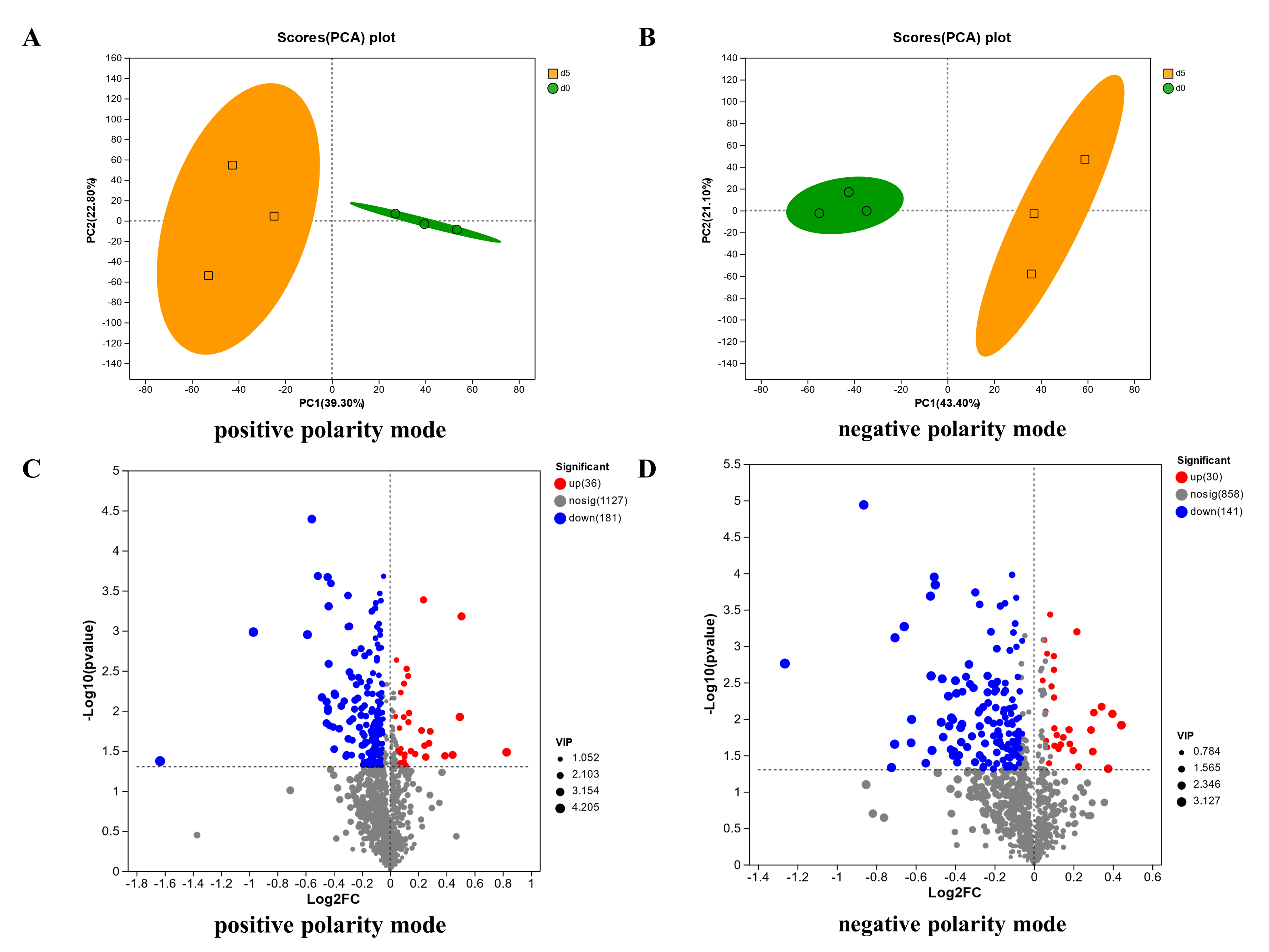

Supplement: Supplementary file 3 — Supplementary Material 3: Figure S3. Overview of metabolomic data between infected and healthy P. koraiensis. (A) Principal component analysis of infected and healthy P. koraiensis during LC-MS/MS analysis in positive mode. (B) Principal component analysis of infected and healthy P. koraiensis during LC-MS/MS analysis in negative mode. (C) A global view of the number of differentially abundant metabolites between infected and healthy P. koraiensis in positive polarity mode. (D) A global view of the number of differentially abundant metabolites between infected and healthy P. koraiensis in negative polarity mode. [file 12870_2025_6192_MOESM3_ESM.tif]

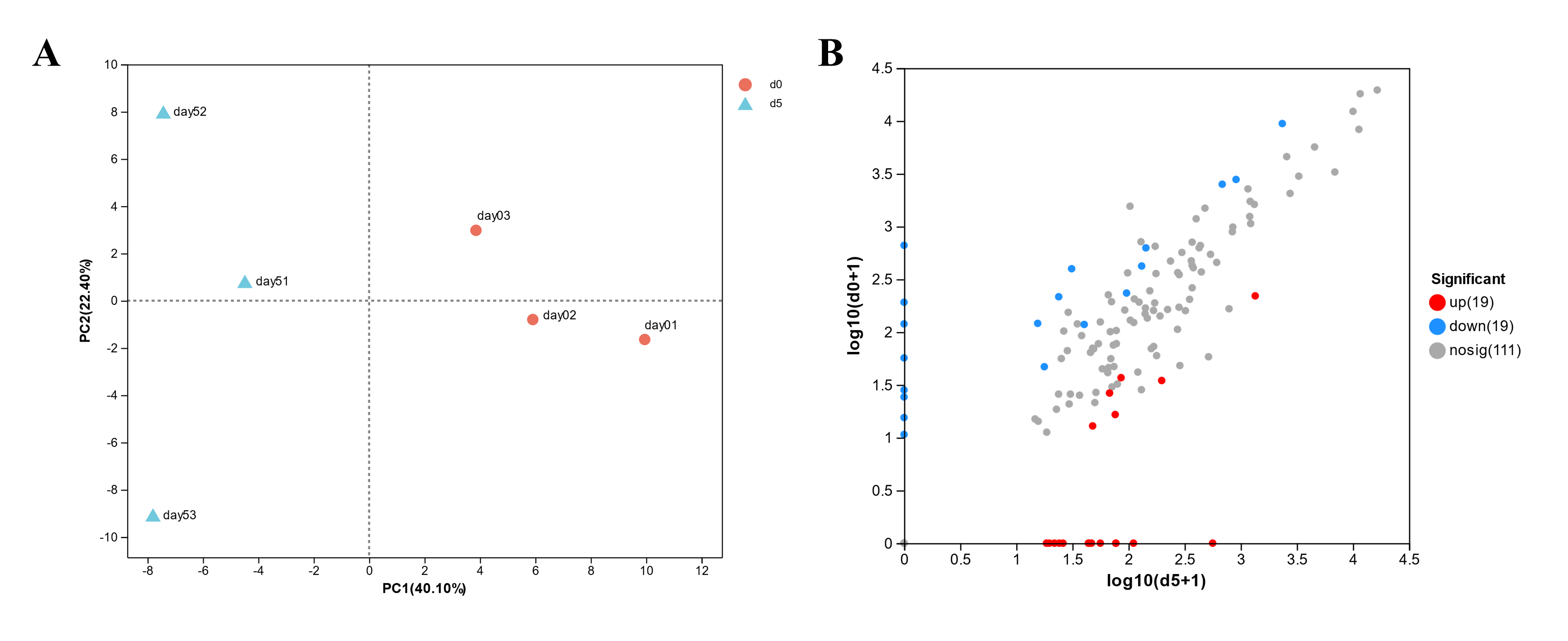

Supplement: Supplementary file 4 — Supplementary Material 4: Figure S4. Overview of proteomic data of infected and healthy P. koraiensis. (A) Principal component analysis of the proteomic data of infected and healthy P. koraiensis. (B) Global view of the number of differentially abundant proteins in infected and healthy P. koraiensis. [file 12870_2025_6192_MOESM4_ESM.tif]
